# Detection of protozoan and helminth parasites in concentrated wet mounts of stool using a deep convolutional neural network

**DOI:** 10.1128/jcm.01062-25

**Published:** 2025-10-21

**Authors:** Blaine A. Mathison, Katie Knight, Jill Potts, Ben Black, John F. Walker, Falon Markow, Amy Wood, Dustin Bess, Ken Dixon, Brian Cahoon, Weston Hymas, Marc Roger Couturier

**Affiliations:** 1 Institute for Clinical and Experimental Pathology, ARUP Laboratories 549001, Salt Lake City, Utah, USA; 2 Department of Pathology, University of Utah School of Medicine 12348 , Salt Lake City, Utah, USA; 3 Institute for Research and Innovation in Diagnostic and Precision Medicine, ARUP Laboratories 33294, Salt Lake City, Utah, USA; 4 Techcyte Inc., Orem, Utah, USA; Mayo Clinic Minnesota, Rochester, Minnesota, USA

**Keywords:** parasite, parasitology, helminth, protozoan, artificial intelligence, convolutional neural network

## Abstract

Comprehensive diagnosis of gastrointestinal parasites is largely reliant on traditional stool microscopy, despite gains in molecular diagnostics. Wet-mount examinations remain a significant challenge for traditional microscopy, digital microscopy, and artifical intelligence (AI). We developed and validated a deep convolutional neural network (CNN) model that provides highly sensitive detection and presumptive classification of enteric parasites. Twenty-seven different parasites were trained on a CNN model using a wide diversity of 4,049 unique parasite-positive specimens (determined by traditional microscopy) collected in the USA, Europe, Africa, and Asia. Model validation was performed with a unique holdout set. In clinical validation, AI correctly detected 250/265 positive specimens (94.3% agreement) and 94/100 negative specimens (94.0%) before discrepant resolution. AI also detected 169 additional organisms from the validation specimens that were not previously identified. These additional detections underwent further discrepant analysis to adjudicate the results by scan review and microscopy. After resolution and inclusion of newly defined true positives and false positives, the positive agreement was 472/477 (98.6%). Negative agreement was variable by organism, ranging from 91.8% to 100%. A relative limit of detection study was performed comparing AI to three technologists of varying experience using serial dilutions of specimens containing *Entamoeba*, *Ascaris*, *Trichuris*, and hookworm. AI consistently detected more organisms and at lower dilutions of parasites than humans, regardless of the technologist’s experience. The use of AI for wet-mount analysis is highly sensitive and detects considerably more organisms than traditional microscopy alone. The use of AI simplifies the parasitology workflow and reduces the reliance on traditional microscopy.

**IMPORTANCE:**

Gastrointestinal parasite ova and parasite (O&P) detection from stools is a manual, labor-intensive method requiring highly trained personnel. This testing has been largely unchanged in 100 years, with the exception of minor improvements in processing and fixation techniques. O&Ps are performed worldwide on millions of stool specimens a year, making any improvements in the process highly impactful. Digital slide imaging and artificial intelligence were recently established tools by our laboratory for improving permanent trichrome stain interpretation. This work builds on that breakthrough and describes the first comprehensive wet-mount AI model development and validation. Improved diagnostic yield, analytical sensitivity, and precision were demonstrated in this work through full clinical laboratory validation studies, including a specimen collection sourced from four continents and a diversity of fixatives and preparation techniques. This work represents the completion of a groundbreaking effort to bring parasite screening into the technological age.

## INTRODUCTION

The ongoing global burden of parasitic diseases continues to demand sensitive laboratory testing for appropriate detection and management of these organisms. With increased global travel and recent mass human migration/displacement events, lab testing is even more critical today for laboratories outside of regions traditionally endemic for many parasites. Despite advancements in diagnostic methods such as antigen detection, nucleic-acid amplification tests (NAATs), and genomic-based assays in both clinical microbiology and parasitology specifically, manual microscopy remains the gold standard for the detection and identification of most parasites (1). Unfortunately, traditional parasitology faces numerous challenges in the clinical laboratory, such as the length of time it takes to be competent or proficient in the discipline and, in turn, maintaining competency when dealing with staff turnover. It is also laborious, time-consuming, and interpretation is largely subjective. Being a sub-discipline of applied zoology, parasitology requires a deep understanding of anatomy, biology, taxonomy, and epidemiology, adding additional challenges to maintaining competency (2).

Traditional stool microscopy for parasitology involves the ova-and-parasite (O&P) exam, which consists of a concentrated wet mount for detecting helminth eggs/larvae and protozoan cysts, and a permanent stained smear for detecting protozoan cysts and trophozoites; however, in many parts of the world, only wet mounts are performed. Because of progress seen in areas such as infrastructure, sanitation, health care, and hygiene and personal habits, fewer parasite cases are seen in developed countries. Those infections that are seen are often in immigrants from, or people who traveled to, areas with a higher incidence of endemic parasitic diseases. As such, laboratorians can spend considerable time manually examining stool specimens only to see positive rates of 2%–5%. Over time, this can lead to low job satisfaction, fatigue, and “burnout,” which can lead to diagnostic errors (1).

Since traditional parasitology still relies on the recognition and identification of morphological features, digital microscopy and artificial intelligence provide an excellent opportunity to assist in the screening and detection of clinical specimens for parasites. Proof-of-concept technologies have been described, but there are very few commercially available assays available for detecting and identifying gastrointestinal parasites.

The work described here represents a functional extension of a 2020 study (1); this time, it analyzed the wet mount portion of the O&P exam using a novel detection model and an additional scanner system. The goal of this study was to demonstrate improved sensitivity for the detection of parasites in true-positive specimens while reducing the need to review true-negative specimens on a microscope.

## MATERIALS AND METHODS

### Classification of categories for model development

Training classes were identified to comprehensively detect necessary targets conventionally reported in concentrated wet mounts of stool. Thirty classes were created for training: (i) *Blastocystis* species, (ii) *Entamoeba* species non-*hartmanni* cysts, (iii) *Entamoeba* species non-*hartmanni* trophozoites, (iv) *Iodamoeba buetschlii* cysts, (v) *Endolimax nana* cysts, (vi) Miscellaneous Small Protozoans (*I. buetschlii* trophozoites, *E. nana* trophozoites, *Dientamoeba fragilis* trophozoites, *Entamoeba hartmanni* cysts, and trophozoites), (vii) *Giardia duodenalis* cysts, (viii) *G. duodenalis* trophozoites, (ix) *Chilomastix mesnili* cysts, (x) *C. mesnili* trophozoites, (xi) *Balantioides coli* cysts, (xii) *B. coli* trophozoites, (xiii) *Cystoisospora belli* oocysts, (xiv) *Cyclospora* species oocysts, (xv) *Ascaris lumbricoides* fertile mamillated eggs, (xvi) *A. lumbricoides* infertile mamillated eggs, (xvii) *Trichuris trichiura* eggs, (xviii) hookworm/*Trichostrongylus* eggs, (xix) *Enterobius vermicularis* eggs, (xx) *Strongyloides* species L1 larvae, (xxi) *Paracapillaria philippinensis* eggs, (xxii) fish tapeworm (*Diphyllobothrium*-complex) eggs, (xxiii) *Taenia* species eggs, (xxiv) *Rodentolepis nana* eggs, (xxv) *Hymenolepis diminuta* eggs, (xxvi) *Schistosoma mansoni* eggs, (xxvii) *Schistosoma japonicum* eggs, (xxviii) *Fasciola* species/*Fasciolopsis buski* eggs, (xxix) *Paragonimus* species eggs, and (xxx) *Clonorchis sinensis*/*Opisthorchis* species eggs. See Table 1 for the number of unique scans and examples per class used in training. For clinical validation, classes were combined based on the species or morphological similarities, resulting in 25 classes (see below under *Clinical laboratory validation*).

**TABLE 1:** Total number of unique scans per class, and total number of examples per class used for training the model

Category (class)	No. of unique scans per class	No. of unique examples per class
*Blastocystis* species	131	1,968
*Entamoeba* species non-*hartmanni* cysts	168	1,401
*Entamoeba* species non-*hartmanni* trophozoites	116	1,650
*E. nana* cysts	92	1,964
*I. buetschlii* cysts	61	1,474
Miscellaneous small protozoans^a^	182	3,116
*G. duodenalis* cysts	83	3,611
*G. duodenalis* trophozoites	51	1,782
*C. mesnili* cysts	63	1,438
*C. mesnili* trophozoites	28	1,546
*B. coli* cysts	133	466
*B. coli* trophozoites	65	1,398
*Cyclospora* species oocysts	23	1,813
*C. belli* oocysts	17	1,296
*A. lumbricoides* fertile eggs	134	983
*A. lumbricoides* infertile eggs	183	894
*T. trichiura* eggs	207	1,037
Hookworm/*Trichostrongylus* eggs	421	1,268
*Strongyloides* species L1 larvae	33	1,215
*P. philippinensis* eggs	201	1,044
*E. vermicularis* eggs	246	796
Fish tapeworm eggs (*Diphyllobothrium*-complex)	333	987
*Taenia* species eggs	97	1,283
*R. nana* eggs	58	1,018
*H. diminuta* eggs	224	1,023
*S. mansoni* eggs	262	1,088
*S. japonicum* eggs	719	989
*Paragonimus* species eggs	116	1,044
*Fasciola* species/*F. buski* eggs	219	504
*Clonorchis sinensis*/*Opisthorchis* species eggs	28	1,095
Background/artifacts	1,778	38,339

^a^ *D. fragilis* trophozoites, *E. nana* trophozoites, *I. buetschlii* trophozoites, *E. hartmanni* cysts, and trophozoites.

### Specimen collection, preparation, and scanning

Due to the numerous targets required for training and validation, specimens were procured from a variety of sources. Most of the protozoans and several of the more common helminths were acquired from residual clinical specimens and residual proficiency specimens reported from the ARUP parasitology laboratory in accordance with our approved IRB #7275. Some of the less common organisms were purchased from Microbiologics (St. Cloud, MN) or provided by colleagues from Mayo Medical Laboratory, Rochester, MN, USA (Dr. Bobbi S. Pritt), the Centers for Disease Control and Prevention, Atlanta, GA, USA (Mr. Henry S. Bishop), University of the Philippines Manila, Manila, Philippines (Dr. Vicente Y. Belizario, Jr. and Taggart G. Saio), Recteur Université Libreville, Norde, Libreville, Gabon (Dr. Rodrigue N. Mintsa), Southern Cross University, Lismore, Australia (Dr. Polydor N. Mutombo), Catholic University of Health and Allied Sciences, Mwanza, Tanzania (Dr. Humphrey D. Mazigo), Universidad Complutense de Madrid, Madrid, Spain (Dr. Francisco Ponce-Gordo), and Usmanu Danfodiyo University, Sokoto, Nigeria (Dr. Aliyu Mahmuda). All specimens are of human origin except for the *Fasciola hepatica* from Africa and the *B. coli* from Spain, which came from cattle and captive orangutans, respectively. Stool specimens used for model training and validation were collected in a variety of fixatives used in clinical parasitology laboratories, including 10% formalin, TOTAL-FIX, EcoFix, Alcorfix, Unifix, and sodium acetate-acetic acid-formalin. All specimens were processed per standard stool procedures, including vertical/centrifugal filtration, gravity filtration, or ethyl acetate concentration.

Slides were prepared by mixing 15 µL of concentrated stool with 20 µL of mounting medium consisting of 1:1 mixture of Lugol’s iodine and 10% glycerol in phosphate-buffered saline on a 75 × 25 mm glass microscope slide. The mounting medium both reduces drying of the specimens and prevents movement of the coverslip, while highlighting internal features of protozoans without making helminth eggs as dark as traditional Lugol’s iodine (Supplemental Data Section 1, Tables S1 through S3 and Fig. S1 through S3). Stool specimens that were unable to be pipetted were sampled using a wooden applicator stick and mixed into 20 µL of the mounting medium. The stool-mounting medium slurry was covered with a 22 × 22 mm glass coverslip on a defined region of the slide using a template to ensure that the proper scanning location was achieved. Scanning for development was performed using four scanners: (i) SpectralHT2 and (ii) SpectralM-Pro scanners (Pramana Inc., Cambridge, Massachusetts), (iii) Hamamatsu NanoZoomer S360 (Hamamatsu Photonics K.K., Hamamatsu City, Japan), (iv) Grundium Ocus40 (Grundium Oy, Tampere, Finland), all equipped with 20× objectives. The central 20 × 20 mm area of the coverslip was scanned at 34×, 42×, and 40× (0.283, 0.230, and 0.250 microns per pixel, respectively). Scans are saved as OME-TIFF, NDPI, or TIFF and transferred to a folder for upload to Techcyte (Techcyte Inc., Orem, UT). Each scan contains 14 prevalence regions evenly spaced over the entire scan area. Acceptability criterion for a valid scan was defined as having achieved at least 11 of the 14 prevalence regions in focus.

### Initial classification and labeling

The first scanned images were manually reviewed for organisms. Exemplars were labeled by creating a box snugly around the organism and assigning the correct organism’s class. These first scanned images were the basis for the initial training sets used to train the model. Additional examples from these and additional slides were detected by the software using active learning, whereby earlier versions of a trained model propose new examples, and the results were verified or refuted by expert analysis as supervised learning. New examples that represented the expected class and showed acceptable focus for training were confirmed. Any organisms that were flagged by the software but misidentified (class confusion; e.g., the software detects *C. mesnili* but classifies it *G. duodenalis*) were reclassified. Organisms that were correctly detected, but deemed inadequate for training (e.g., unacceptable focus), were labeled as “unknown” so the software would not detect them again in future runs. Artifacts that were detected and classified as organisms were labeled “background” or had unique anti-classes created for them and trained as background, so future models would be less likely to make the same false predictions. This process was repeated until the software failed to find new examples or until at least 1,000–2,000 examples were detected and confirmed. It was important not to have a high percentage of examples coming from one or a few slides, so the features of the organisms and background would not be biased toward higher-count slides. Labeling was performed using the Techcyte cloud-hosted data storage and web interface.

### Training a deep convolutional neural network

**Fig 1:**
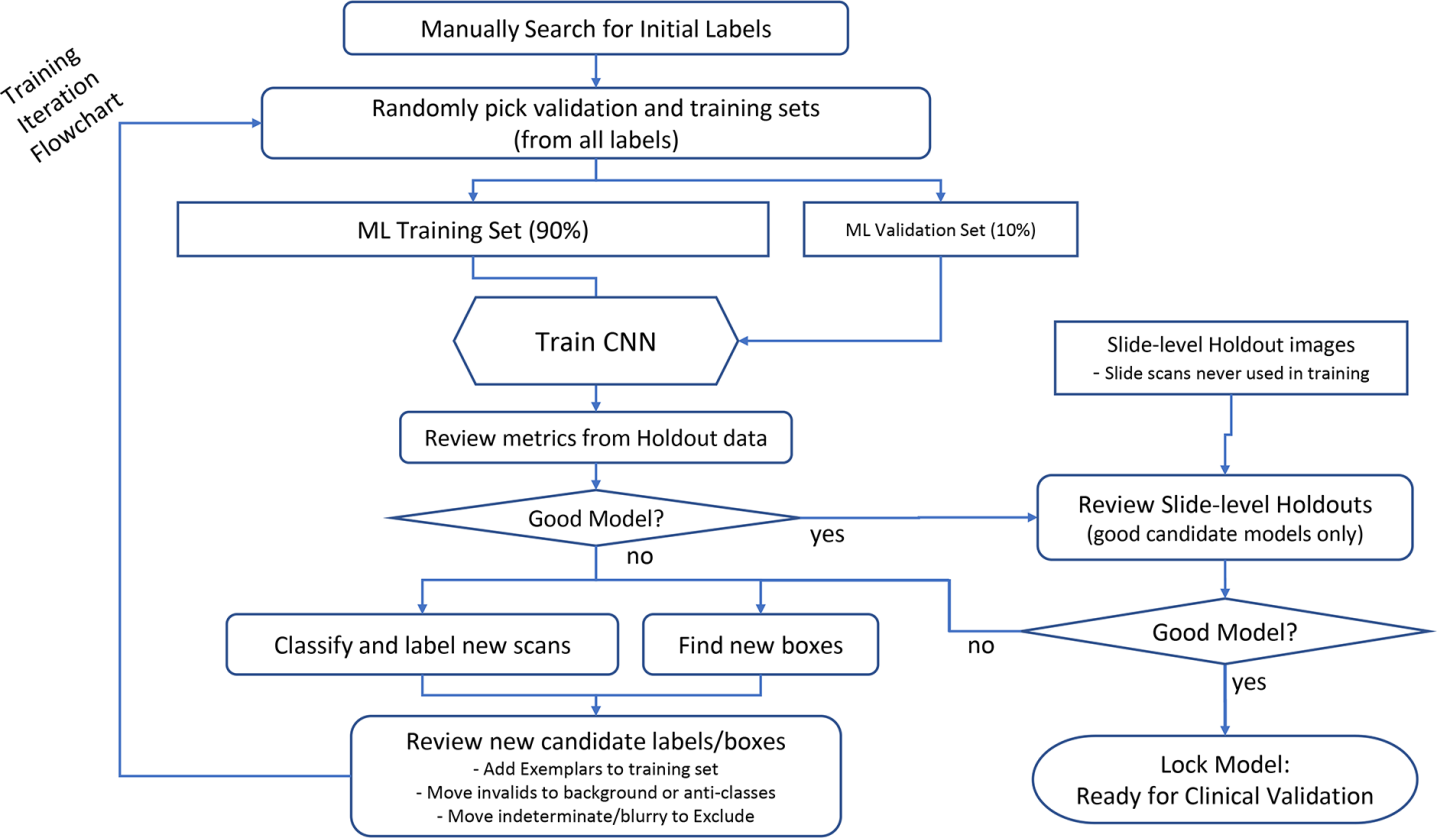
Flow chart summary of training iterations and hold-out validation for CNN. ML, machine learning.

Numerous training runs were performed during development, and the output metrics were used to analyze progress (Fig. 1). The training data set comprises all labels from every organism class plus labels representing background. A randomly arranged 768 × 768-pixel image was cropped to encompass a labeled box at pre-processing time. Ten percent of the labels for each class were randomly selected and used as convolutional neural network (CNN) training validation data before the training began. This was used to measure progress during and after training. Augmentation is applied to scenes from the training data set, which are generated dynamically for each epoch. The model is based on the YOLOv3 architecture (3), a fully convolutional, anchor-based, single-pass object detection architecture. Each model trained during the development process was initialized from the pretrained weights trained on the standard ImageNet (4) and COCO (5) data sets. Most hyperparameters were taken from the YOLO3 architecture, with the following modifications to optimize for fine-tuning on top of the pretrained model. A batch size of size was used due to the relatively large training scene size taking up too much graphics processing unit (GPU) memory. An exponential decayed learning schedule was used, starting at 1 × 10^−4^ and ending at 1 × 10^−5^. Scenes with rare object types were over-sampled to balance the loss between the classes. Training continued for 12 epochs, taking 15 hours in total on a Nvidia A10 GPU.

### Image processing/augmentation for training

Since training labels are placed sparsely on large whole slide images, during pre-processing time, random 768 × 768 crops are taken around each label in such a way that the entire label’s box is fully included but is otherwise random so that the model learns to make predictions anywhere on an image. For each training epoch, this crop and its associated labels are randomly augmented with rotations/flips and hue/saturation color augmentations. Since the whole slide image in the training set is not comprehensively labeled, the crops typically include some unlabeled pixel data adjacent to the training labels. To avoid bias against this unlabeled data, model predictions located in unlabeled areas of one of these crops are masked out and not penalized in the loss function.

### Image processing for model training (inference)

Once the whole slide image is uploaded, the trained CNN is shown a sequence of 4,032 × 2,016-pixel scenes to look for parasites for which it creates a labeled image box. This large scene size, chosen for processing efficiency, is modified after training without fine-tuning or special techniques, made possible due to the use of a translation-invariant fully convolutional CNN architecture. These scenes are overlapped horizontally and vertically by 428 px (~110 µm). Larger parasites will still be mostly included in a scene, with at least 55% of a 650 px (~160 µm) object being included in one scene, even if it appears right in the corner of four tiles. Model predictions with a confidence score below a cutoff threshold are rejected and not displayed (Supplemental Sections 3 and 4). After the entire image is processed, duplicates are removed with non-max suppression and labeled image boxes are grouped by class and sorted by decreasing confidence for display.

### Holdout data

A holdout set is a distinct set of labeled data not used during model training. Entirely different slides have a set of expert-confirmed labels. For each class, these labels are spread evenly between all scanners and at least 2–5 specimens to ensure that different scanners and specimens are represented in metrics in a balanced manner. To calculate per-object metrics such as per-object recall, these expert labels are matched against the model’s predictions, with the scan being processed in inference mode (large tiles with overlaps). A match is defined by a predicted, ground-truth pair with the same class and an intersection over union score of at least 0.5. Mismatched boxes are treated as false positives or false negatives in precision-recall calculations. This matching provides a set of metrics independent of training data, which are used for hyperparameter tuning and data development prioritization throughout the model development process (Supplemental Section 2 and 4). These metrics are also used to assist final threshold selection. All per-object metrics of model performance displayed here will use these holdout results.

### Confidence score threshold determination

**Fig 2:**
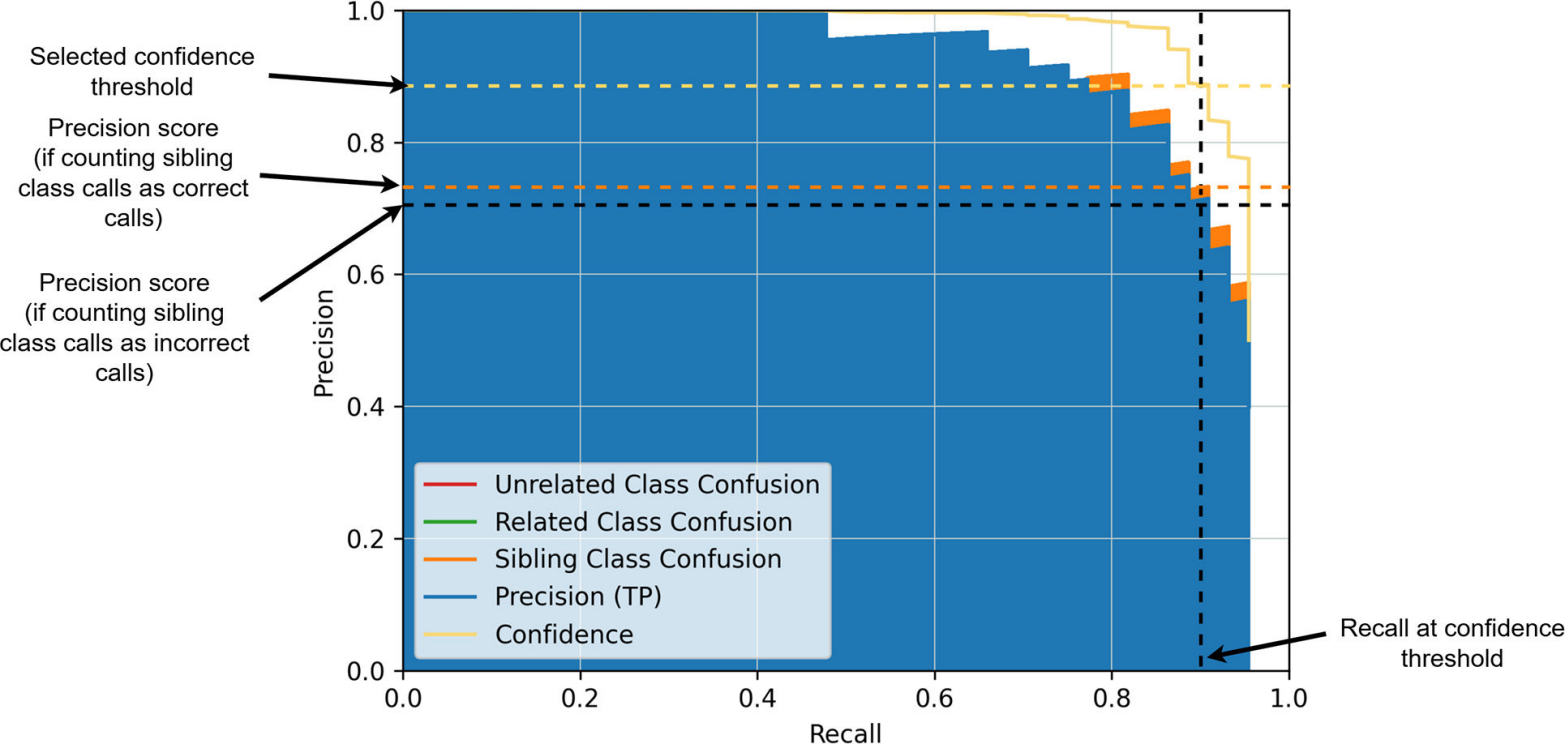
Precision-recall curve example. Each point along the curve corresponds to a precision/recall tradeoff on the selected by a particular confidence threshold. A single trade-off point is highlighted in the figure. NOTE* (Machine Learning Definitions used. Precision: akin to Positive-Predictive Value or the ratio of true positives over the sum of true positives plus false positives [e.g., what portion of the objects found are correct]. Recall – akin to sensitivity or the ratio of true positives over the sum of true positives and false negatives [e.g. what fraction of true objects are detected].)

Confidence scores are predicted alongside the box and label during CNN inference. Lower confidence scores indicate a weaker match to the predicted class. Before any filtering, the CNN produces millions of predictions per slide, so confidence score thresholds are picked to filter down these predictions and ensure that only reasonably strong predictions are displayed. Confidence scores are unscaled, and class-specific thresholds were determined by examining precision-recall (PR) curves on the holdout data. A PR curve is a plot that visualizes the tradeoff between precision and recall in the holdout data at every possible confidence threshold (see Fig. 2 for an example). It offers actionable insight into threshold behavior over a large set of slides and objects. Confidence thresholds were chosen manually based on these plots to maximize recall without sacrificing too much precision. Though the training data set was identical for all models, independent models and confidence thresholds were selected separately for each scanner vendor, yielding three models with three sets of thresholds, one for Pramana, Hamamatsu, and Grundium. The difference between the models and thresholds was small; however, we did observe model performance and confidence scores to differ distributionally on our holdout metrics based on scanner vendor, due to differences in brightness, color, optics, postprocessing, etc. Identifying a single model and thresholds for all scanners was suboptimal.

#### Clinical laboratory validation

Clinical validation was performed on the Pramana SpectralHT2 scanner and the AI model selected for that scanner as mentioned above.

### Scan area design

In clinical practice, the entire 22 × 22 mm area of the coverslip is read with 10× and 40× objectives. For clinical validation, the center 20 × 20 mm area of the coverslip was scanned at 0.283 microns per pixel (34× equivalent magnification) requiring approximately 6 minutes per scan.

### Slide (organism) classification algorithm

For clinical validation, several classes trained individually during development were combined based on the species or otherwise shared morphological features that might lead to frequent class confusion. The final 25 categories displayed in the user interface of the software are as follows: (i) *G. duodenalis*, (ii) *Entamoeba* species, (iii) *C. mesnili* cysts, (iv) *E. nana* cysts, (v) *I. buetschlii* cysts, (vi) Miscellaneous Small Protozoans (trophozoites of *D. fragilis*, *E. nana*, *I. buetschlii*, *C. mesnili*, and cysts and trophozoites of *E. hartmanni*), (vii) *Blastocystis* species, (viii) *B. coli*, (ix) *Cyclospora* species, (x) *C. belli*, (xi) *A. lumbricoides*, (xii) *T. trichiura*, (xiii) hookworm/*Trichostrongylus*, (xiv) fish tapeworm, (xv) *Taenia* species, (xvi) *E. vermicularis*, (xvii) *Strongyloides* species, (xviii) *R. nana*, (xix) *H. diminuta*, (xx) *S. mansoni*, (xxi) *S. japonicum*, (xxii) *P. philippinensis*, (xxiii) *Paragonimus* species, (xxiv) *Fasciola* species/*F. buski*, and (xxv) *C. sinensis*/*Opisthorchis* species.

### Specimen collection and preparation

For the accuracy portion of the initial validation, 10 concentrates (none of which were used previously for model development) were scanned and analyzed for each of the following 26 organisms: (i) *D. fragilis*, (ii) *G. duodenalis*, (iii) *E. nana*, (iv) *Cyclospora* species, (v) *E. hartmanni*, (vi) *Entamoeba* sp. non-*hartmanni*, (vii) *C. mesnili*, (viii) *I. buetschlii*, (ix) *Blastocystis* species, (x) *C. belli*, (xi) *B. coli*, (xii) *A. lumbricoides*, (xiii) *T. trichiura*, (xiv) hookworm/*Trichostrongylus*, (xv) fish tapeworm, (xvi) *Taenia* species, (xvii) *E. vermicularis*, (xviii) *Strongyloides* species, (xix) *R. nana*, (xx) *H. diminuta*, (xxi) *S. mansoni*, (xxii) *S. japonicum*, (xxiii) *P. philippinensis*, (xxiv) *Paragonimus* species, (xxv) *Fasciola* species/*F. buski*, and (xxvi) *C. sinensis*/*Opisthorchis* species. Several specimens were known to contain multiple species. Because of the rarity of some of these organisms in clinical practice, some positive specimens were diluted into multiple unique negative stool specimens of the same fixative to achieve a minimum of 10 unique validation specimens. During validation, if any specimens were reclassified as true negatives or had issues that prevented scanning, an attempt was made to acquire additional specimens to supplement the specimen set to 10. All classes except *S. japonicum* were able to reach 10 positive accuracy specimens. In addition, 100 unique specimens previously reported as negative for parasites in our lab were scanned and analyzed. During validation, if a specimen dried and prevented it from being scanned, new specimens were acquired to reach a minimum of 100.

Slides were prepared as above and scanned in batches of 5–10 to prevent drying. It should be noted that other factors could affect drying, such as specimens that are grainy or have other large particulate matter or excess mucus. Particulate matter can also affect focus, resulting in the scanner homing in on the wrong plane.

### Accuracy

Accuracy specimens were defined as having a “ground truth” at the initiation of the studies based on the previous standard of care test results reported by the originating laboratory. The specimens were not retested in our laboratory before enrollment in these studies. Accuracy was performed using a combination of slide-level agreement and organism-level agreement (allowing for class confusion) as an accuracy metric. An initial agreement was performed with 10 “ground truth” positives for each organism and 100 “ground truth” negative specimens. *Blastocystis* had 15 accuracy specimens to account for 10 random positives at 3+ or 4+ prevalence and five positives at a lower prevalence of 1+ or 2+. Due to the unexpected number of previously undetected or undocumented organisms in several specimens, discrepant analysis required organism-level agreement analysis for equitable and complete data analysis. Clinical validation criteria were set such that agreement needed to reach 90% or more for satisfactory performance on an organism level (this metric was predetermined before any validation studies occurred by our laboratory; see Supplemental Data section 5 for the Accuracy workflow).

### Discrepant resolution studies

If the accuracy studies did not match “ground truth,” a discrepant analysis was required to establish the resolved truth. This involved preparing a new slide from the initial specimen and reviewing it manually. This was performed when a parasite was detected by the software but not expected based on “ground truth” or when expected organisms were not detected. In the latter example, the scan was scrutinized for the intended organism before a new slide was prepared. If the intended organism was not detected by the software and not found by review of the scan but was found on microscopic review of a new preparation, then a new slide was prepared and scanned. A true positive was defined as a slide containing parasites that the software detected (whether it was an intended organism or an additional organism) that was confirmed upon discrepant analysis. A true negative was defined as a slide that did not contain parasites and for which the model generated no labels or few labels that did not trigger a manual examination of the slide. A false positive was defined as a slide that did not contain parasites but for which the model detected putative parasites that subsequently necessitated manual review of the slide to confirm. A false negative was defined as a scan in which parasites were not detected by the model, but parasites were found on manual review of the digital scan by a human.

### Precision

Precision studies were performed using a stool containing *Taenia*, a stool containing *G. duodenalis*, and a negative stool. Specimens were scanned three times on the same day to best simulate intra-run precision. The same specimens were then prepared fresh and scanned on 2 additional days for a total of 3 days of inter-run precision. Slide-level agreement was evaluated, and the total organisms detected were documented for each scan, inclusive of true organism and class confusion.

### Limit of detection

To test the limit of detection (LoD), a specimen containing *A. lumbricoides* eggs, *T. trichiura* eggs, *Entamoeba coli* cysts and trophozoites, and low-level hookworm eggs was serially diluted in stool fixed in 10% formalin that had been evaluated as negative by concentrated wet mount, trichrome stain, and ultraviolet microscopy. The specimen was serially diluted to 1:512, resulting in 11 aliquots, including the neat (undiluted). Three technologists were selected based on their parasitology expertise, with approximately 8 years, 4–5 years, and less than 1 year, respectively. The same dilution series was used for all three technologists. The 11 aliquots were randomized so that the technologist was not reading them in the order they were diluted. A slide was prepared for the technologist using 20 µL of mounting medium and 15 µL of specimen. The technologist was allowed to read the slide per normal procedure and given approximately 5 minutes to complete the read. If the technologist was still reading at minute 5, they were allowed to finish so as not to make them feel rushed. They were asked to report what they observed at the level possible for a wet mount. They were also asked to note the rarity of organisms seen or semi-quantify them if few were observed. After the technologist read the slide, the slide was immediately scanned, so the software was analyzing the same preparation read by the technologist. The results of the technologists’ analyses and software were documented. Total numbers provided for the scans account for both correct classifications and class confusion.

## RESULTS

### Holdout set results

Full precision-recall tradeoff curves for all classes on the Pramana holdout data are displayed in Supplemental Section 2, with an explanation of how to read them in Fig. 2. As discussed in Materials and Methods, these plots, along with dry runs, were used to select specific thresholds for the Pramana model. Selected thresholds for all classes are displayed in Supplemental Section 3. After threshold selection, per-object recall on the Pramana specific holdout was greater than 80% per-object recall except for some small protozoans: *Blastocystis* spp. (70%), *E. nana* cysts (68%), *G. duodenalis* cysts and trophozoites (0.79%/0.67% respectively), miscellaneous small protozoans (0.67%, class includes *D. fragilis* trophozoites, *E. nana* trophozoites, *I. buetschlii* trophozoites, and *E. hartmanni* cysts and trophozoites). Additionally, precision for every class was also greater than 80% except for some of the small protozoans: *Blastocystis* spp. (75%) and *C. mesnili* cyst (79%). Full results of holdout precision and recall after thresholding are shown in Supplemental Data 4Supplemental Data 4. These holdout results were reviewed as proof of concept before going forward with the study.

### Accuracy

Initial accuracy was determined for each organism trained in the AI model. Table 2 shows the initial slide-level agreement for the validation study set based on the ground truth of enrolled specimens. Initial agreement revealed that each organism reached >90% agreement (19 at 100% and 3 at 90%), with the exception of *Chilomastix*, *Trichuris*, and *S. japonicum. S. japonicum* and *Chilomastix* each failed to detect 5 and 4 positive specimens, respectively. The model failed to detect two *Trichuris*-positive specimens. The overall positive agreement was 94.3% (250/265). The initial negative agreement was over 90% for all classes, with few false positives identified by the model for any particular class. Overall negative agreement was 94% (94/100).

**TABLE 2:** Accuracy (based on “ground truth”) results for each class on the initial valid scan^*a*^

Organism	Positive accuracy	Negative accuracy
*D. fragilis*	9/10 (90%)	99/100 (99%)
*G. duodenalis*	10/10 (100%)	100/100 (100%)
*E. nana*	10/10 (100%)	99/100 (99%)
*Cyclospora* species	10/10 (100%)	100/100 (100%)
*E. hartmanni*	10/10 (100%)	99/100 (99%)
*Blastocystis* species	15/15 (100%)	100/100 (100%)
*C. mesnili*	6/10 (60%)	99/100 (99%)
*Entamoeba* species, non-*hartmanni*	10/10 (100%)	99/100 (99%)
*I. buetschlii*	10/10 (100%)	99/100 (100%)
*C. belli*	10/10 (100%)	100/100 (100%)
*B. coli*	10/10 (100%)	100/100 (100%)
*A. lumbricoides*	9/10 (90%)	100/100 (100%)
*T. trichiura*	8/10 (80%)	100/100 (100%)
Hookworm/*Trichostrongylus* sp.	10/10 (100%)	100/100 (100%)
Fish tapeworm (*Diphyllobothrium*-complex)	9/10 (90%)	100/100 (100%)
*Taenia* species	10/10 (100%)	100/100 (100%)
*E. vermicularis*	10/10 (100%)	100/100 (100%)
*Strongyloides* species	9/10 (90%)	100/100 (100%)
*R. nana*	10/10 (100%)	100/100 (100%)
*H. diminuta*	10/10 (100%)	100/100 (100%)
*S. mansoni*	10/10 (100%)	100/100 (100%)
*P. philippinensis*	10/10 (100%)	100/100 (100%)
*Paragonimus* species	10/10 (100%)	100/100 (100%)
*Fasciola* sp./*F. buski*	10/10 (100%)	100/100 (100%)
*Clonorchis*/*Opisthorchis* spp.	10/10 (100%)	100/100 (100%)
*S. japonicum*	5/10 (50%)	100/100 (100%)
Total agreement	250/265 (94.3%)	94/100 (94%)

^*a*^ Additional (secondary) expected organisms and extra organisms found by the scan or discrepant analysis are not considered here (see Table 3).

### Accuracy, post-discrepant analysis for resolved truth

Discrepant resolution revealed a significant number of previously unknown positive organisms in the validation study set, as well as additional false positive results that were identified during the subsequent discrepant resolution. One hundred three scans detected at least one putative unexpected organism that required manual confirmation. In 84 of these (81.6%), at least one of the additional organisms was confirmed by manual microscopy or image analysis (when the prevalence was too rare to be found after preparing a second smear) to establish “resolved truth.” After resolving truth based on the discrepant resolution criteria defined in the methods (see above Materials and Methods, Accuracy), the total number of true-positive specimens increased by nearly twofold (265 initial, vs 477 resolved). The overwhelming number of multiply infected specimens also made slide-level analysis impractical, so organism-level agreement analysis was employed after resolution testing. After resolution testing, several classes contained 20 or more true positive specimens, including: *Blastocystis*, *Giardia*, *E. nana*, *Entamoeba* sp., *Iodamoeba*, *Trichuris*, hookworm*/Trichostrongylus,* and miscellaneous small protozoa (Table 3). Only *Chilomastix* failed to reach >90% positive agreement after discrepant resolution studies (81%, 13/16). The total positive agreement was 98.9% across all organisms (472/477). The AI model identified an additional 193 true positive organisms compared to the initial accuracy results after resolution of discrepancies. Due to the complicated nature of reclassifying false positives in mixed infections, overall negative agreement could not be easily calculated in aggregate and was determined at an organism level rather than slide level. As such, each organism had adjusted negative accuracy (Table 3). All organism classes reached 90% negative agreement with microscopy (after the described resolution analysis).

**TABLE 3:** Total agreement (representing “resolved truth”) per organism, including additional organisms detected by the model or during discrepant analysis^*d*^

Organism	Positive	Negative
*D. fragilis*	11/11 (100%)	102/103 (99%)
*G. duodenalis*	26/26 (100%)	103/104 (99%)
*E. nana*	21/21 (100%)	102/103 (99%)
*Cyclospora* species	11/11 (100%)	103/104 (99%)
*E. hartmanni*	12/12 (100%)	102/103 (99%)
*Blastocystis* species	61/61 (100%)	103/110 (94%)
*C. mesnili*	13/16 (81%)	102/104 (98%)
*Entamoeba* species, non-*hartmanni*	49/49 (100%)	102/112 (91%)
*I. buetschlii*	20/20 (100%)	102/104 (98%)
*C. belli*	10/10 (100%)	103/103 (100%)
*B. coli*	13/13 (100%)	103/103 (100%)
*A. lumbricoides*	17/18 (95%)	103/104 (99%)
*T. trichiura*	30/30 (97%)	103/103 (100%)
Hookworm/*Trichostrongylus* sp.	26/26 (100%)	103/103 (100%)
Fish tapeworm (*Diphyllobothrium*-complex)	10/10 (100%)	103/103 (100%)
*Taenia* species	10/10 (100%)	103/103 (100%)
*E. vermicularis*	11/11 (100%)	103/103 (100%)
*Strongyloides* species	17/18 (94%)	103/104 (99%)
*R. nana*	13/13 (100%)	103/103 (100%)
*H. diminuta*	10/10 (100%)	103/103 (100%)
*S. mansoni*	10/10 (100%)	103/103 (100%)
*P. philippinensis*	10/10 (100%)	103/103 (100%)
*Paragonimus* species	10/10 (100%)	103/103 (100%)
*Fasciola* sp./*F. buski*	10/10 (100%)	103/103 (100%)
*Clonorchis*/*Opisthorchis* spp.	10/10 (100%)	103/103 (100%)
*S. japonicum*	9/9^*a*^ (100%)	103/103 (100%)
Misc. Small Protozoans^*b*^	22/22 (100%)	102/111 (92%)
Total Agreement	472/477 (98.9%)	Variable by target^*c*^

^*a*^ In an effort to obtain 10 true positives for *S. japonicum*, four additional specimens were acquired. Three out of four were positive for *S. japonicum* by scanning. The fourth was negative, so another specimen was acquired that was also negative. After which, no positive specimens remained to test. The final positive agreement for *S. japonicum* was 9/9.

^*b*^ These are protozoan trophozoites that could not be further identified without a cyst stage present or a corresponding trichrome smear, and for the negative values, objects flagged as miscellaneous protozoans that turned out to be artifacts or host cells.

^*c*^ Specimens identified to contain additional organisms were not enrolled as additional negative accuracy specimens and instead were only considered for the target organism identified by AI. As such, overall negative accuracy numbers did not increase for each organism when discrepant resolution was performed. Negative accuracy may increase for specific organisms if false positives were determined after discrepant resolution.

^*d*^ Specimens that were read as completely negative by scan and manual read were added as true negatives for every class.

### Precision-clinical microbiology analysis

Precision of the AI model was determined using a single negative specimen and two unique specimens containing *Taenia* or *Giardia*. Each positive organism and negative stool showed 100% inter- and intra-run precision (Supplemental data Section 6). The total number of organisms detected in each scan/preparation was variable, as would be expected in a heterogeneous stool specimen.

### Limit of detection

A specimen containing *E. coli*, *Trichuris*, *Ascaris*, and hookworm was used to challenge the relative limit of detection between the AI model and a technologist. For *E. coli*, the software consistently detected organisms to 1:64, with the technologists confidently detecting the organism from 1:4 to 1:8 (Table 4). Technologists B and C detected rare *E. coli* again at 1:128 and 1:32, respectively. The software detected more objects than the technologists did for this organism. For dilutions 1:1, 1:2, 1:4, and 1:8, Technologist A reported only two organisms at 1:1 and the other three dilutions as “rare.” In comparison, the software flagged 99 organisms at 1:1, 75 at 1:2, 25 at 1:4, and 10 at 1:8, accounting for both correct classifications and class confusion. Similarly, Technologist B indicated *E. coli* was rare at 1:2, although the software detected 70 organisms (Table 4).

**TABLE 4:** Relative LoD comparison of each technologist and the corresponding scan for *Entamoeba* spp. non-*hartmanni*^*a*^

	*Entamoeba coli* (as class representative)					
	Series A (8 years exp.)		Series B (<1 year exp.)		Series C (3.5–4 years exp.)	
Dilution	Software	Technologist	Software	Technologist	Software	Technologist
Neat	Detected (*n* = 185)	Detected	Detected (*n* = 261)	Detected	Detected (*n* = 141)	Detected
1:1	Detected (*n* = 99)	Detected (*n* = 2)	Detected (*n* = 101)	Detected	Detected (*n* = 120)	Detected
1:2	Detected (*n* = 75)	Detected (rare)	Detected (*n* = 70)	Detected (rare)	Detected (*n* = 44)	Detected
1:4	Detected (*n* = 25)	Detected (rare)	Detected (*n* = 40)	Detected	Detected (*n* = 26/27)	Detected
1:8	Detected (*n* = 10)	Detected (*n* = 2)	Detected (*n* = 6)	Not detected	Detected (*n* = 16)	Detected
1:16	Detected (*n* = 2/3)	Not detected	Detected (*n* = 3)	Not detected	Detected (*n* = 2)	Not Detected
1:32	Detected (*n* = 2)	Not detected	Detected (*n* = 2)	Not detected	Detected (*n* = 1)	Detected (suspicious)
1:64	Detected (*n* = 1)	Not detected	Detected (*n* = 1/2)	Not detected	Detected (*n* = 3)	Not detected
1:128	Not detected	Not detected	Not detected	Detected (possible, rare)	Not detected	Not detected
1:256	Not detected	Not detected	Detected (*n* = 1?)	Not detected	Not detected	Not detected
1:512	Not detected	Not detected	Not detected	Not detected	Not detected	Not detected

^*a*^ Grey shade indicates that the values were not detected.

The software detected *T. trichiura* consistently at 1:8, with additional detections at 1:64 and 1:256 for Series A. These detections included both correct classifications and class confusion (in two series, a single egg at 1:8 was misclassified as *P. philippinensis*). The technologists detected *T. trichiura* consistently at 1:2, and one technologist detected it at 1:4 and 1:64. The software did not detect any eggs at 1:64. Manual review of the scan did not reveal the presence of *T. trichiura* eggs; the eggs may have been out of the scan area (e.g., along the extreme edge of the coverslip), or out of focus (Table 5).

**TABLE 5:** Relative LoD comparison of each technologist and the corresponding scan for *T. trichiura*^*c*^

	*T. trichiura*					
	Series A (8 years exp.)		Series B (<1 year exp.)		Series C (3.5–4 years exp.)	
Dilution	Software	Technologist	Software	Technologist	Software	Technologist
Neat	Detected (*n* = 49)	Detected	Detected (*n* = 49)	Detected	Detected (*n* = 45)	Detected
1:1	Detected (*n* = 12)	Detected	Detected (*n* = 15)	Detected	Detected (*n* = 22)	Detected
1:2	Detected (*n* = 10)	Detected	Detected (*n* = 16)	Detected	Detected (*n* = 11)	Detected
1:4	Detected (*n* = 1)	Not detected	Detected (*n* = 5)	Detected (rare)	Detected (*n* = 9)	Detected
1:8	Detected (*n* = 1)^*a*^	Not detected	Detected (*n* = 1)^*a*^	Not detected	Detected (*n* = 1)	Not detected
1:16	Not detected	Not detected	Detected (*n* = 1)	Not detected	Not detected	Not detected
1:32	Not detected	Not detected	Detected (*n* = 1)	Not detected	Not detected	Not detected
1:64	Detected (*n* = 1)	Not detected	Not detected	Detected (rare)^*b*^	Not detected	Not detected
1:128	Not detected	Not detected	Not detected	Not detected	Not detected	Not detected
1:256	Detected (*n* = 1)	Not detected	Not detected	Not detected	Not detected	Not detected
1:512	Not detected	Not detected	Not detected	Not detected	Not detected	Not detected

^*a*^ Single *T. trichiura* eggs were misclassified as *P. philippinensis*.

^*b*^ *T. trichiura* could not be found on manual review of the scan; the egg(s) may have been out of the scan area or out of focus of the scan.

^*c*^ Grey shade indicates that the values were not detected.

The software detected *A. lumbricoides* at 1:4 to 1:16 depending on the preparation, with a single egg at 1:256 for Series B. Technologists detected *A. lumbricoides* at 1:2 to 1:8 depending on the preparation, with rare eggs detected at 1:32 (Technologist A) and 1:64 (Technologist B). In both of those latter two instances, the eggs were not detected by the software. The eggs may have been out of the scan area or out of focus. Technologist C did not detect *A. lumbricoides* eggs in the neat preparation, although the software detected 23 eggs (Table 6).

**TABLE 6:** Relative LoD comparison of each technologist and the corresponding scan for *A. lumbricoides*^*b*^

	*A. lumbricoides*					
	Series A (8 years exp.)		Series B (<1 year exp.)		Series C (3.5–4 years exp.)	
Dilution	Software	Technologist	Software	Technologist	Software	Technologist
Neat	Detected (*n* = 23)	Detected	Detected (*n* = 23)	Detected	Detected (*n* = 23)	Not detected
1:1	Detected (*n* = 3)	Detected	Detected (*n* = 7)	Detected	Detected (*n* = 12)	Detected
1:2	Detected (*n* = 5)	Detected	Detected (*n* = 1/2)	Detected	Detected (*n* = 5)	Detected
1:4	Detected (*n* = 1)	Not detected	Detected (*n* = 2)	Detected	Detected (*n* = 1)	Not detected
1:8	Detected (*n* = 2)	Not detected	Detected (*n* = 1)	Detected (rare)	Not detected	Not detected
1:16	Detected (*n* = 1)	Not detected	Not detected	Not detected	Not detected	Not detected
1:32	Not detected	Detected (*n* = 1)^*a*^	Not detected	Not detected	Not detected	Not detected
1:64	Not detected	Not detected	Not detected	Detected (possible, rare)^*a*^	Not detected	Not detected
1:128	Not detected	Not detected	Not detected	Not detected	Not detected	Not detected
1:256	Not detected	Not detected	Detected (*n* = 1)	Not detected	Not detected	Not detected
1:512	Not detected	Not detected	Not detected	Not Detected	Not Detected	Not detected

^*a*^ *A. lumbricoides* could not be found on manual review of the scan; the egg(s) may have been out of the scan area or out of focus of the scan.

^*b*^ Grey shade indicates that the values were not detected.

The software detected hookworm at 1:1 or 1:4 depending on the preparation, with an additional single egg at 1:64 for Series C. Technologists A and B detected hookworm at 1:1 but not in the neat specimen. Technologist C detected hookworm only at 1:64 and no other dilutions, including the neat specimen (Table 7).

**TABLE 7:** Relative LoD comparison of each technologist and the corresponding scan for hookworm^*a*^

	Hookworm					
	Series A (8 years exp.)		Series B (<1 year exp.)		Series C (3.5–4 years exp.)	
Dilution	Software	Technologist	Software	Technologist	Software	Technologist
Neat	Detected (*n* = 2)	Not detected	Detected (*n* = 2)	Not detected	Detected (*n* = 4)	Not detected
1:1	Detected (*n* = 2)	Detected (*n* = 1)	Detected (*n* = 2)	Detected (rare)	Detected (*n* = 1)	Not detected
1:2	Not detected	Not detected	Not detected	Not detected	Detected (*n* = 1)	Not detected
1:4	Not detected	Not detected	Not detected	Not detected	Detected (*n* = 1)	Not detected
1:8	Not detected	Not detected	Not detected	Not detected	Not detected	Not detected
1:16	Not detected	Not detected	Not detected	Not detected	Not detected	Not detected
1:32	Not detected	Not detected	Not detected	Not detected	Not detected	Not detected
1:64	Not detected	Not detected	Not detected	Not detected	Detected (*n* = 1)	Detected
1:128	Not detected	Not detected	Not detected	Not detected	Not detected	Not detected
1:256	Not detected	Not detected	Not detected	Not detected	Not detected	Not detected
1:512	Not detected	Not detected	Not detected	Not detected	Not detected	Not detected

^*a*^ Grey shade indicates that the values were not detected.

## DISCUSSION

Detection of parasites in stool specimens still relies heavily on labor-intensive manual microscopy. Although our work previously using AI for trichrome stains has shown a vast improvement in workflow and sensitivity, the second component of an O&P (wet mount) remains manual (1). In many parts of the world, only the wet mount portion is performed. As such, a tool that allows for sensitive screening of parasites from liquid stool is sorely needed in the global marketplace. There are a few commercially available assays using artificial intelligence or machine learning for the detection of intestinal parasites. In the 2024 study by Boonyoung et al. in Thailand, the FA280 AI + Feces Analyzer (Sichuan Orienter Biotechnology Co. Ltd., Chengdu, China) showed 100% agreement when images captured by the system were scrutinized by an expert parasitologist but dropped to 75.5% when analyzed by the system’s software. When compared to manual microscopy using 800 formalin-fixed stools, the system demonstrated 95% agreement, with parasites being detected in 440 specimens using the FA280 accompanied by expert review of the images and parasites being detected in 468 specimens by manual microscopy (6). A 2021 study in Korea specifically looking at the detection of *C. sinensis* using AVE-562 stool analyzer (AVE Science and Technology Co., Ltd., Hunan, China) demonstrated only 36.4% sensitivity, 100% specificity, 100% positive predictive value, and 73.1% negative predictive value, although the study used a rather small sample size (*n* = 30) (7). The Techcyte Fecal Ova and Parasite Detection system (Techcyte Inc., Orem, Utah, USA) uses a convolutional neural network to detect common intestinal protozoans, white blood cells (WBCs), and red blood cells (RBCs) in trichrome-stained stool specimens. Our laboratory previously validated the Techcyte system using 87 positive specimens and 106 negative specimens, demonstrating 98.88% positive slide-level agreement and 98.11% slide-level negative agreement (1).

This study aimed to develop and validate an AI model capable of sensitive screening of gastrointestinal (GI) parasites, while optimizing sensitivity over organism-level specificity. This was based on the premise that, despite trained morphologists becoming less and less common in the clinical laboratory (2), the ability for high-resolution images to be aggregated and presented to an adequately trained medical microbiologist could overcome the deficit of highly specialized and expert-level clinical parasitology technologists. In other words, a competent laboratorian can recognize an organism when presented multiple images, rendering this AI model as a screen-out tool for negatives and a presumptive-positive detection tool that requires a human for ultimate arbitration of truth (akin to the trichrome model described previously). Having a model that rarely, if ever, fails to detect a true positive specimen would represent a dramatic improvement in O&P performance.

The model described in this study was designed to detect all conventional GI protozoan and helminth parasites. The organisms trained in this study represented a global collaboration and network of experts. An immediate advantage of this model is the ability to detect organisms that were previously limited to special stains (e.g., modified acid-fast, modified safranin) in most labs, such as the coccidia, *Cyclospora*, and *Cystoisospora*. Many labs either do not screen for these critically important organisms or rely on the special stains, which recently were shown to be less sensitive than UV microscopy (8). A laboratory could conceivably eliminate the special stains or use the AI model to augment and complement the detection of these organisms.

The accuracy of the model described in this validation study of the wet-mount model was quite striking and surprising to an extent. Specimens enrolled in this study contained a documented “ground truth” based on standard lab testing results. As was shown in Table 3, the model was able to detect nearly 50% more organisms than ”ground truth.” This discrepancy and “resolved truth” were not unique to specimens from a single study collection site but rather dispersed across the specimen set. As these archived results would have represented standard-of-care test results for most of the specimens, this increase in diagnostic yield achieved by AI is dramatic. After discrepant resolution, several of the classes that failed to meet the initial criteria of 90% agreement did, in fact, surpass that metric of standard of care microscopy when “resolved truth” was established. One conclusion from these data would be that the standard of care microscopy fails to meet the performance standard of an AI wet-mount screening tool for most organisms. This can be confidently concluded since an expert parasitologist manually confirmed each of these additional detections. It is clear from this study that integration of an AI model for wet-mount examinations will likely lead to the detection of multi-parasite infections more often than manual microscopy alone.

The non-pathogenic flagellate *C. mesnili* failed to reach 90% positive agreement after resolution. This organism is small and difficult to separate from background debris and artifacts. As such, the training data resulted in too many or too few organisms being correctly detected. Due to the excellent performance characteristics of the trichrome stain for this class, we feel that this target performance can be overcome by using the trichrome stain. In geographic regions that only perform a wet mount, this organism will not consistently be detected but is non-pathogenic and may be an acceptable tradeoff.

Several false negative results were documented, which would have clinical implications. One example was a known positive *Strongyloides* specimen that had a preponderance of rare, heavily degraded, and out-of-focus organisms. A manual review of the specimen by an expert confirmed the presence of *Strongyloides* but commented on the difficulty to confirm based on the few organisms and the relatively poor state of the organisms. Regardless, manual examination did detect the presence when AI simply failed to detect these degraded forms as *Strongyloides*. A future goal for this model could be to bolster the data set with additional examples of heavily degraded organisms to ensure near-perfect detection of this organism. Unfortunately, training on heavily degraded or out-of-focus organisms can also have unwanted consequences, such as false-positive detection of plant fibers and other look-alikes. An important counterbalance to this false negative is the additional eight true positive *Strongyloides* specimens that were detected by AI, which were not previously detected by manual microscopy.

An additional class that revealed a dramatic increase in diagnostic yield was *Trichuris*. The initial study set included 10 positive specimens, of which only eight were detected on initial analysis due to two preparations not having eggs present (confirmed by manual review of the scan). However, after full resolution of the study set, “resolved truth” reflected that 30 specimens contained *Trichuris*, essentially a 66% increased diagnostic yield. Several of these were very low-prevalence specimens, while others were simply missed or not reported by the previous standard of care testing. This increased detection capability for *Trichuris* was reflected in the limit of detection studies as well (see below).

The relative LoD described previously for trichrome AI (1) was largely replicated by the results of these studies, albeit with a bit more variability in the degree of difference between organisms and technologists. The overall sensitivity of the AI model, pairing the accuracy results with the relative LoD studies, showed better sensitivity for AI. Some organisms, such as hookworms, were in very low numbers and were not detected even in the neat specimen. In fact, none of the scans of neat specimens detected more than five eggs (Table 7). The relative comparison to technologists was also variable based on experience, which is likely extrapolatable to most labs. In fact, technologist variability is an intrinsic limitation of any lab, whereas a locked-down AI model and a well-maintained scanner is largely controllable and reproducible. An additional observation of the LoD studies is random detection of an organism at low levels, sometimes out of order in the dilution series (e.g., failure to detect an egg at 1:2 and 1:4 dilution, followed by detection at 1:8). This was seen with humans and AI; however, an important note is that failure to detect the neat and initial dilutions was only observed for humans and never for AI. The most important conclusion to consider, though, is that this AI model represents a tool for the clinical lab, and presumptive detections will simply aid the technologist in determining true identifications with higher frequency. One potential unforeseen complication of this increased performance is how a laboratory will handle a scan detection of a rare egg, which cannot subsequently be detected by manual exam. Laboratories will have to determine whether ultimate identification can be achieved simply by an image alone or whether confirmation by microscopy is required.

This study has several strengths to highlight. First, the training, study, and hold-out sets represent the largest and most comprehensive organism collections in contemporary parasitology literature. Unique specimens were used for each stage of development (training, hold-out, and validation). The size and diversity of the study sets are further strengthened by the fact that the specimens were not obtained at a single site but instead represent a diversified collection of fixatives conventionally used worldwide, different conventional concentration techniques used across various regions, and sourced from patients across different geographies and cultures. This last point is critical, as this broad origin of stool allows for a diversity of backgrounds given the different global dietary habits. This resulted in background diversity that strengthens the overall training set. Lastly, the training set was further diversified with scan data obtained from numerous scanners, which not only strengthens the model but also allows the model to be ported across multiple scanners available in the commercial marketplace.

This study has several practical limitations. First, the discrepant resolution was complicated and required multiple re-preps due to organisms not included in “ground truth,” as well as challenges with low-prevalence specimens and approaching the practical limit of detection of conventional microscopy. Furthermore, it was difficult to obtain some organisms due to the global rarity of their diseases. This resulted in some classes (e.g., *S. japonicum*) failing to reach the desired 10 positive specimens. Likewise, there are at least two organisms that are not trained or detected in this model. The first is *Cryptosporidium*, while despite some laboratories using wet mount to detect this parasite, this is not considered good practice and therefore was not trained on the wet-mount model. Detection is better achieved by antigen detection or NAAT (9, 10). The second organism group is the Acanthacephala. The acanthacephalans are rare zoonotic parasites in humans. As recently reviewed, most cases are identified by detection of adult worms in stool and not by detection of eggs in stool (11). As such, acanthacephalan eggs will not specifically be detected by this model. Pilot testing using *Moniliformis* as a representative of the Acanthacephala revealed that the model identified *Moniliformis* eggs as *Ascaris*; however, with only one specimen to test this inclusion, the actual sensitivity for that detection cannot be determined. An additional limitation is that for some specimens in the validation set that were obtained from other countries, it is not known whether the specimens were always screened for other organisms when assigning “ground truth,” vs part of a specific screening initiative only (e.g., *Schistosoma*). As such, we cannot definitively state that all unexpected positive results for the complete study set were unique detections not previously known, albeit the majority were specimens reported per standard of care. The scanner used in this study only scanned 20 × 20, and not the entire 22 × 22 coverslip, which could conceivably lead to missed organism detections. Lastly, the specimens in this study did not have corresponding trichrome stains to comprehensively compare the total findings with.

In conclusion, we present the development and validation of an AI model to augment in the detection of GI parasites from fixed fecal specimens as part of a comprehensive O&P examination. The model showed increased diagnostic yield compared to conventional microscopy across a highly diversified study set derived from across the world. AI showed excellent positive and negative agreement with manual microscopy, with improved sensitivity overall. Prospective studies are underway to evaluate the real-time diagnostic yield of this technology in our laboratory pre and post integration, as well as a workflow analysis for time and cost considerations. Other study sites using different commercial scanners with the models developed in this work are currently underway to provide evidence of the portability of performance.

## AFTER EPUB

*[This article was published on 21 October 2025 with an error in a genus name. The name was corrected in the current version, posted on 21 October 2025.]*

## SUPPLEMENTAL MATERIAL

Supplemental material jcm.01062-25-s0001.docx Supplemental methods, data, and Figures S1 to S3, and Tables S1 to S3.

ASM does not own the copyrights to Supplemental Material that may be linked to, or accessed through, an article. The authors have granted ASM a non-exclusive, world-wide license to publish the Supplemental Material files. Please contact the corresponding author directly for reuse.
