# Supplementary material for: Detection of protozoan and helminth parasites in concentrated wet mounts of stool using a deep convolutional neural network
Source: J Clin Microbiol. 2025 Oct 21;63(11):e01062-25. doi: 10.1128/jcm.01062-25 (PMC12607898; doi:10.1128/jcm.01062-25)
Supplement: Supplemental material — Supplemental methods, data, and Figures S1 to S3, and Tables S1 to S3. [file jcm.01062-25-s0001.docx]

**Supplemental Data.**

Detection of protozoan and helminth parasites in concentrated wet mounts of stool using a deep convolutional neural network.

Blaine A. Mathison^a,d^, Katie Knight^b^, Jill Potts^c^, Ben Black^c^, John F. Walker^c^, Falon Markow^c^, Amy Wood^c^, Dustin Bess^c^, Ken Dixon^c^, Brian Cahoon^c^, Weston Hymas^a^, Marc Roger Couturier^a,d^

**1. Creation of a Mounting Medium**

Various reagents were initially tested, including NP-40, Tween 20, Triton X, and glycerol. The chemicals were mixed at various concentrations with phosphate-buffered saline (PBS) and then mixed 1/1 with Lugol’s iodine. In the end, glycerol at a 10% concentration in PBS (for a total of 5% when mixed 1/1 with Lugol’s iodine) provided the best visual clarity while preserving drying time and binding to the coverslip.

A. Initially 50 specimens in various fixatives were tested. They were observed for upwards of three hours and their drying times were recorded (see **Tables S1-3, Figures S1-3**). The mean drying time was 134 minutes and the median drying time was 148 minutes. The mean drying time for formalin-fixed stools was 122 minutes while for alcohol-based fixatives the mean drying time was 146 minutes.

B. The second set of experiments was to compare the drying times for 10 specimens under the following situations: 1) no mounting medium, 2) Lugol’s iodine (only), 3) 50/50 glycerol/Lugol’s mix. Interestingly, the data did not seem to favor the presence of a mounting medium or the kind of mounting medium. The average times were as follows:

- No mounting medium: 133 minutes
- Lugol’s (only): 120 minutes
- Lugol’s/glycerol: 107 minutes

c. The third set of experiments was to take two specimens from the experiments in ‘b’ (above) and test them each 10 times. One specimen was chosen that had not dried at three hours (Specimen 2). The other specimen had dried the fastest (Specimen 4). The goal was to see how much variability there would be within the same specimen. For Specimen 2, the average drying time was 119.5 minutes. None of the 10 replicates lasted the entire 3 hours as it did during the earlier experiments (b, above). For Specimen 4, the average drying time was 34.9 minutes. Overall, it appears that a variety of factors may affect drying time, including, but not limited to, fixative, user technique, ambient temperature, ambient humidity, and physical characteristics of the sampling of stool. Lugol’s iodine/glycerol mixture was employed during development and validation to aid in image sharpness (e.g. highlighting internal features of protozoans without making helminth eggs too dark), viscosity of the specimen for scanning (e.g. preventing movement of the coverslip), and improve drying times. There are inherent factors that could at any time affect drying and focus, such as grainy specimens or specimens that contain larger than normal amounts of mucus, even with an improved medium and the best practices.

**Table S1.**
Raw data for 50 specimens in various fixatives.

| Specimen | Fixative | Date | Room Temp. (C) | Amb. Humidity | Start Time (H:M:S) | Time Dry (H.M.S) | Total time (H:M:S) | notes | Total Mins |
| --- | --- | --- | --- | --- | --- | --- | --- | --- | --- |
| 1 | 10% Formalin | 23-Jan-24 | 23.5 | 16.50% | 0:00:00 | 2:44:55 | 2:44:55 |  | 165 |
| 2 | 10% Formalin | 23-Jan-24 | 23.5 | 16.50% | 0:00:55 | 3:00:00 | 2:59:05 |  | 180 |
| 3 | 10% Formalin | 23-Jan-24 | 23.5 | 16.50% | 0:01:45 | 1:24:46 | 1:23:01 |  | 83 |
| 4 | 10% Formalin | 23-Jan-24 | 23.5 | 16.50% | 0:02:37 | 2:26:20 | 2:23:43 |  | 144 |
| 5 | 10% Formalin | 23-Jan-24 | 23.5 | 16.50% | 0:03:20 | 3:00:00 | 2:56:40 |  | 177 |
| 6 | 10% Formalin | 23-Jan-24 | 23.5 | 16.50% | 0:04:05 | 2:12:10 | 2:08:05 |  | 128 |
| 7 | 10% Formalin | 23-Jan-24 | 23.5 | 16.50% | 0:05:02 | 1:42:40 | 1:37:38 |  | 98 |
| 8 | 10% Formalin | 23-Jan-24 | 23.5 | 16.50% | 0:05:52 | 3:00:00 | 2:54:08 |  | 174 |
| 9 | 10% Formalin | 23-Jan-24 | 23.5 | 16.50% | 0:06:53 | 1:09:15 | 1:02:22 |  | 122 |
| 10 | 10% Formalin | 23-Jan-24 | 23.5 | 16.50% | 0:07:35 | 1:05:37 | 0:58:02 | grainy specimen | 58 |
| 11 | 10% Formalin | 24-Jan-24 | 22.8 | 23% | 0:00:00 | 0:27:32 | 0:27:32 | grainy specimen | 28 |
| 12 | 10% Formalin | 24-Jan-24 | 22.8 | 23% | 0:09:34 | 1:45:00 | 1:35:26 |  | 95 |
| 13 | 10% Formalin | 24-Jan-24 | 22.8 | 23% | 0:02:06 | 1:43:00 | 1:40:54 |  | 101 |
| 14 | 10% Formalin | 24-Jan-24 | 22.8 | 23% | 0:02:45 | 3:00:00 | 2:57:15 |  | 177 |
| 15 | 10% Formalin | 24-Jan-24 | 22.8 | 23% | 0:03:36 | 1:47:30 | 1:43:54 |  | 104 |
| 16 | 10% Formalin | 24-Jan-24 | 22.8 | 23% | 0:05:15 | 2:45:00 | 2:39:45 |  | 160 |
| 17 | 10% Formalin | 24-Jan-24 | 22.8 | 23% | 0:06:05 | 2:45:00 | 2:38:55 |  | 159 |
| 18 | 10% Formalin | 24-Jan-24 | 22.8 | 23% | 0:06:43 | 3:06:43 | 3:00:00 |  | 180 |
| 19 | 10% Formalin | 24-Jan-24 | 22.8 | 23% | 0:07:35 | 2:50:00 | 2:42:25 |  | 162 |
| 20 | 10% Formalin | 24-Jan-24 | 22.8 | 23% | 0:08:24 | 0:43:44 | 0:35:20 |  | 35 |
| 21 | 10% Formalin | 24-Jan-24 | 22.8 | 23% | 0:11:00 | 1:43:00 | 1:32:00 |  | 92 |
| 22 | 10% Formalin | 24-Jan-24 | 22.8 | 23% | 0:11:41 | 2:29:50 | 2:18:09 |  | 138 |
| 23 | 10% Formalin | 24-Jan-24 | 22.8 | 23% | 0:12:45 | 1:47:30 | 1:34:45 |  | 95 |
| 24 | 10% Formalin | 24-Jan-24 | 22.8 | 23% | 0:13:35 | 2:07:40 | 1:54:05 |  | 114 |
| 25 | 10% Formalin | 24-Jan-24 | 22.8 | 23% | 0:14:17 | 1:47:30 | 1:33:13 |  | 93 |
| 26 | Alcorfix | 25-Jan-24 | 23 | 21.80% | 0:00:00 | 3:00:00 | 3:00:00 |  | 180 |
| 27 | Alcorfix | 25-Jan-24 | 23 | 21.80% | 0:00:43 | 1:15:00 | 1:14:17 |  | 74 |
| 28 | Alcorfix | 25-Jan-24 | 23 | 21.80% | 0:01:52 | 3:01:52 | 3:00:00 |  | 180 |
| 29 | Alcorfix | 25-Jan-24 | 23 | 21.80% | 0:02:58 | 0:12:00 | 0:09:02 | grainy specimen | 9 |
| 30 | Alcorfix | 25-Jan-24 | 23 | 21.80% | 0:03:42 | 2:02:15 | 1:58:33 |  | 119 |
| 31 | Alcorfix | 25-Jan-24 | 23 | 21.80% | 0:04:30 | 1:55:00 | 1:50:30 |  | 111 |
| 32 | Alcorfix | 25-Jan-24 | 23 | 21.80% | 0:06:04 | 3:06:04 | 3:00:00 |  | 180 |
| 33 | Alcorfix | 25-Jan-24 | 23 | 21.80% | 0:06:55 | 2:40:00 | 2:33:05 |  | 153 |
| 34 | Alcorfix | 25-Jan-24 | 23 | 21.80% | 0:08:03 | 1:23:34 | 1:15:31 |  | 76 |
| 35 | Alcorfix | 25-Jan-24 | 23 | 21.80% | 0:08:59 | 3:08:59 | 3:00:00 |  | 180 |
| 36 | Alcorfix | 25-Jan-24 | 23 | 21.80% | 0:09:34 | 3:09:34 | 3:00:00 |  | 180 |
| 37 | Alcorfix | 25-Jan-24 | 23 | 21.80% | 0:10:20 | 3:10:20 | 3:00:00 |  | 180 |
| 38 | EcoFix | 30-Jan-24 | 23 | 17.20% | 0:00:00 | 3:00:00 | 3:00:00 |  | 180 |
| 39 | EcoFix | 30-Jan-24 | 23 | 17.20% | 0:00:35 | 3:00:35 | 3:00:00 |  | 180 |
| 40 | EcoFix | 30-Jan-24 | 23 | 17.20% | 0:01:43 | 2:34:00 | 2:32:17 |  | 152 |
| 41 | EcoFix | 30-Jan-24 | 23 | 17.20% | 0:02:41 | 3:02:41 | 3:00:00 |  | 180 |
| 42 | EcoFix | 30-Jan-24 | 23 | 17.20% | 0:03:35 | 1:54:00 | 1:50:25 |  | 110 |
| 43 | EcoFix | 30-Jan-24 | 23 | 17.20% | 0:04:24 | 3:04:24 | 3:00:00 |  | 180 |
| 44 | TotalFix | 30-Jan-24 | 23 | 17.20% | 0:05:55 | 0:48:00 | 0:42:05 |  | 42 |
| 45 | EcoFix | 30-Jan-24 | 23 | 17.20% | 0:06:50 | 3:06:50 | 3:00:00 |  | 180 |
| 46 | TotalFix | 30-Jan-24 | 23 | 17.20% | 0:07:47 | 3:07:47 | 3:00:00 |  | 180 |
| 47 | TotalFix | 30-Jan-24 | 23 | 17.20% | 0:08:43 | 1:56:29 | 1:47:46 |  | 168 |
| 48 | EcoFix | 30-Jan-24 | 23 | 17.20% | 0:10:10 | 3:10:10 | 3:00:00 |  | 180 |
| 49 | EcoFix | 30-Jan-24 | 23 | 17.20% | 0:11:05 | 1:34:00 | 1:22:55 |  | 143 |
| 50 | TotalFix | 30-Jan-24 | 23 | 17.20% | 0:11:49 | 1:34:00 | 1:22:11 |  | 142 |

**Figure S1.**
Total specimen drying times for each of the 50 test specimens.

**Figure S2.**
Box and whisker plot representation of the total specimen drying times for 50 test specimens.


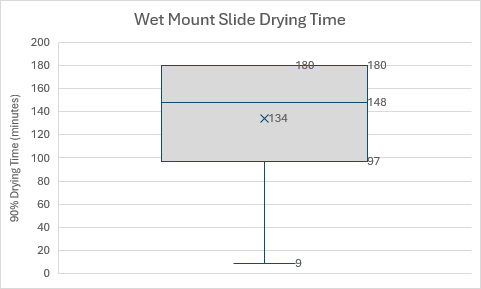


**Figure S3.**
Box and whisker plot for the comparative drying times by fixative comparing alcohol-based with formalin-based fixatives.


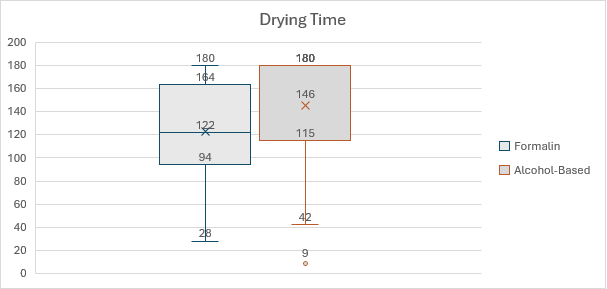


**Table S2.**
Comparison of stool specimens with 1) no mounting medium, 2) Lugol’s iodine (only), and 3) Lugol’s/glycerol mixture.

| Specimen | Fixative | Date | Temp. (C) | Humidity | Times | No MM | Lugol's (only) | Lugol's/glycerol-PBS | Comments |
| --- | --- | --- | --- | --- | --- | --- | --- | --- | --- |
| 1 | Alcorfix | 14-Feb-24 | 23 | 19.90% | Start (h:m) | 0:00 | 0:00 | 0:01 |  |
|  |  |  |  |  | Dry (h:m) | 3:00 | 1:45 | 3:01 |  |
|  |  |  |  |  | Total (m) | 180 | 105 | 180 |  |
| 2 | Formalin | 14-Feb-24 | 23 | 19.90% | Start (h:m) | 0:02 | 0:02 | 0:03 |  |
|  |  |  |  |  | Dry (h:m) | 0:58 | 1:10 | 3:03 |  |
|  |  |  |  |  | Total (m) | 56 | 68 | 180 |  |
| 3 | EcoFix | 14-Feb-24 | 23 | 19.90% | Start (h:m) | 0:04 | 0:04 | 0:04 |  |
|  |  |  |  |  | Dry (h:m) | 1:13 | 3:04 | 0:58 |  |
|  |  |  |  |  | Total (m) | 69 | 180 | 54 |  |
| 4 | Alcorfix | 14-Feb-24 | 23 | 19.90% | Start (h:m) | 0:05 | 0:06 | 0:06 | Grainy specimen |
|  |  |  |  |  | Dry (h:m) | 0:19 | 0:22 | 0:27 |  |
|  |  |  |  |  | Total (m) | 14 | 16 | 27 |  |
| 5 | Formalin | 14-Feb-24 | 23 | 19.90% | Start (h:m) | 0:07 | 0:07 | 0:08 |  |
|  |  |  |  |  | Dry (h:m) | 3:07 | 2:48 | 1:24 |  |
|  |  |  |  |  | Total (m) | 180 | 161 | 76 |  |
| 6 | EcoFix | 14-Feb-24 | 23 | 19.90% | Start (h:m) | 0:09 | 0:09 | 0:10 |  |
|  |  |  |  |  | Dry (h:m) | 3:09 | 2:24 | 0:58 |  |
|  |  |  |  |  | Total (m) | 180 | 135 | 48 |  |
| 7 | Formalin | 14-Feb-24 | 23 | 19.90% | Start (h:m) | 0:10 | 0:11 | 0:11 |  |
|  |  |  |  |  | Dry (h:m) | 3:10 | 3:11 | 3:11 |  |
|  |  |  |  |  | Total (m) | 180 | 180 | 180 |  |
| 8 | Ecofix | 14-Feb-24 | 23 | 19.90% | Start (h:m) | 0:12 | 0:12 | 0:12 |  |
|  |  |  |  |  | Dry (h:m) | 3:12 | 3:12 | 2:16 |  |
|  |  |  |  |  | Total (m) | 180 | 180 | 124 |  |
| 9 | Formalin | 14-Feb-24 | 23 | 19.90% | Start (h:m) | 0:13 | 0:13 | 0:14 |  |
|  |  |  |  |  | Dry (h:m) | 3:13 | 1:30 | 2:09 |  |
|  |  |  |  |  | Total (m) | 180 | 77 | 115 |  |
| 10 | Formalin | 14-Feb-24 | 23 | 19.90% | Start (h:m) | 0:14 | 0:15 | 0:15 |  |
|  |  |  |  |  | Dry (h:m) | 2:09 | 1:55 | 1:32 |  |
|  |  |  |  |  | Total (m) | 115 | 100 | 77 |  |

**Table S3**.

Comparison of two specimens from Table 4. Specimen 4 previously dried the fastest and Specimen 2 did not dry within 3 hours.

| Date | 16-Feb-24 |  |  |  |  |  |  |  |  |  |
| --- | --- | --- | --- | --- | --- | --- | --- | --- | --- | --- |
| Temperature | 23C |  |  |  |  |  |  |  |  |  |
| Amb. Humidity | 17.40% |  |  |  |  |  |  |  |  |  |
|  |  |  |  |  |  |  |  |  |  |  |
| Specimen 2 (FOR) | 1 | 2 | 3 | 4 | 5 | 6 | 7 | 8 | 9 | 10 |
| Set time (h:m) | 0:00 | 0:00 | 0:01 | 0:01 | 0:02 | 0:02 | 0:03 | 0:04 | 0:04 | 0:05 |
| Dry time (h:m) | 3:00 | 1:57 | 2:04 | 2:23 | 2:13 | 1:47 | 1:57 | 1:41 | 1:52 | 1:23 |
| Total (m) | 180 | 117 | 123 | 142 | 131 | 105 | 114 | 97 | 108 | 78 |
|  |  |  |  |  |  |  |  |  |  |  |
| Specimen 4 (ALC) | 1 | 2 | 3 | 4 | 5 | 6 | 7 | 8 | 9 | 10 |
| Set time (h:m) | 0:07 | 0:07 | 0:08 | 0:08 | 0:08 | 0:09 | 0:09 | 0:10 | 0:10 | 0:11 |
| Dry time (h:m) | 0:40 | 0:45 | 0:53 | 0:36 | 0:31 | 0:36 | 0:52 | 0:52 | 0:45 | 0:46 |
| Total (m) | 33 | 38 | 45 | 28 | 23 | 27 | 43 | 42 | 35 | 35 |

**2. Final precision-recall (PR) curves for each class and aggregated PR curve**

Aggregated PR curve including results from all classes:


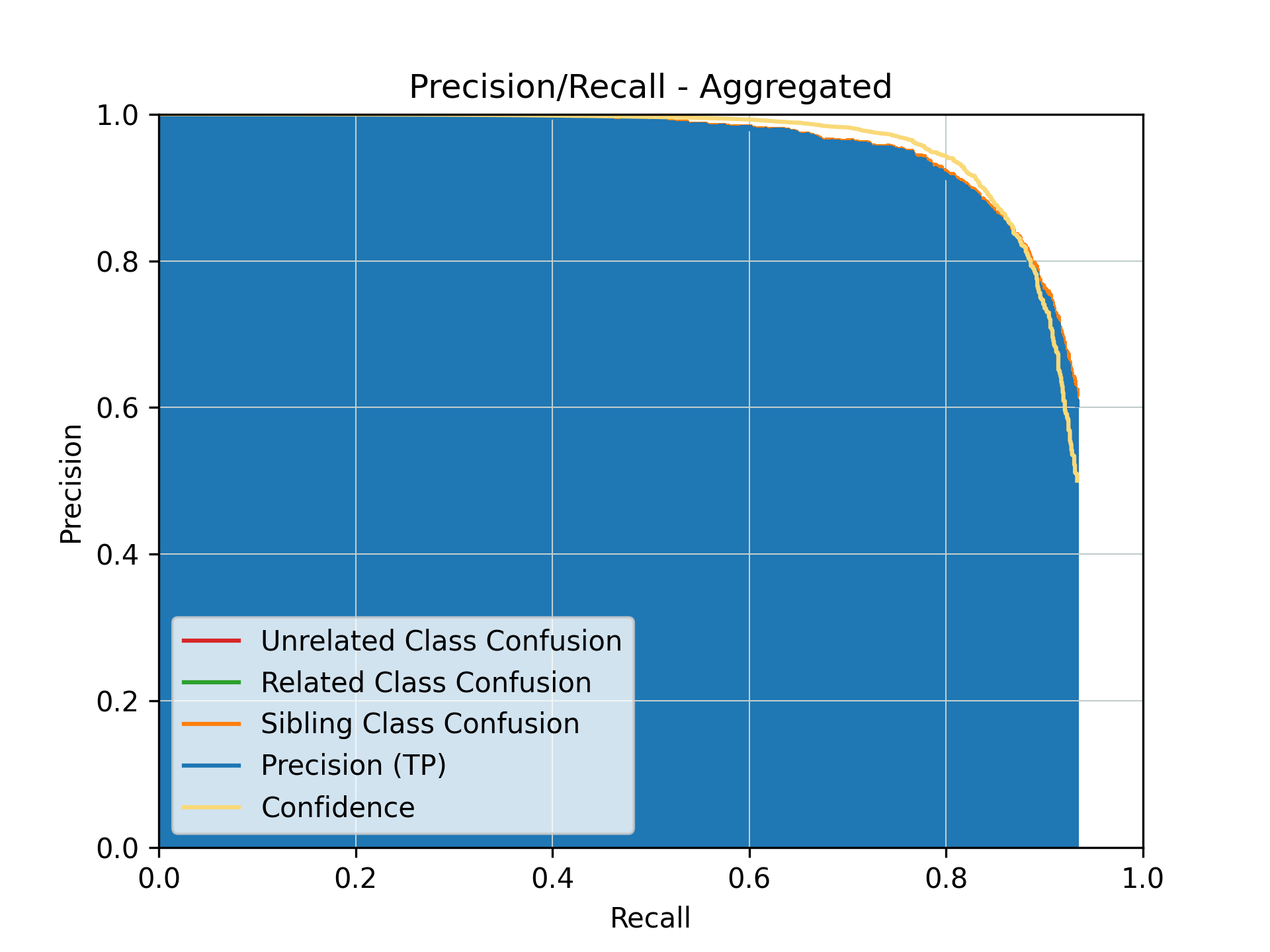


Precision-recall curves for each individual class


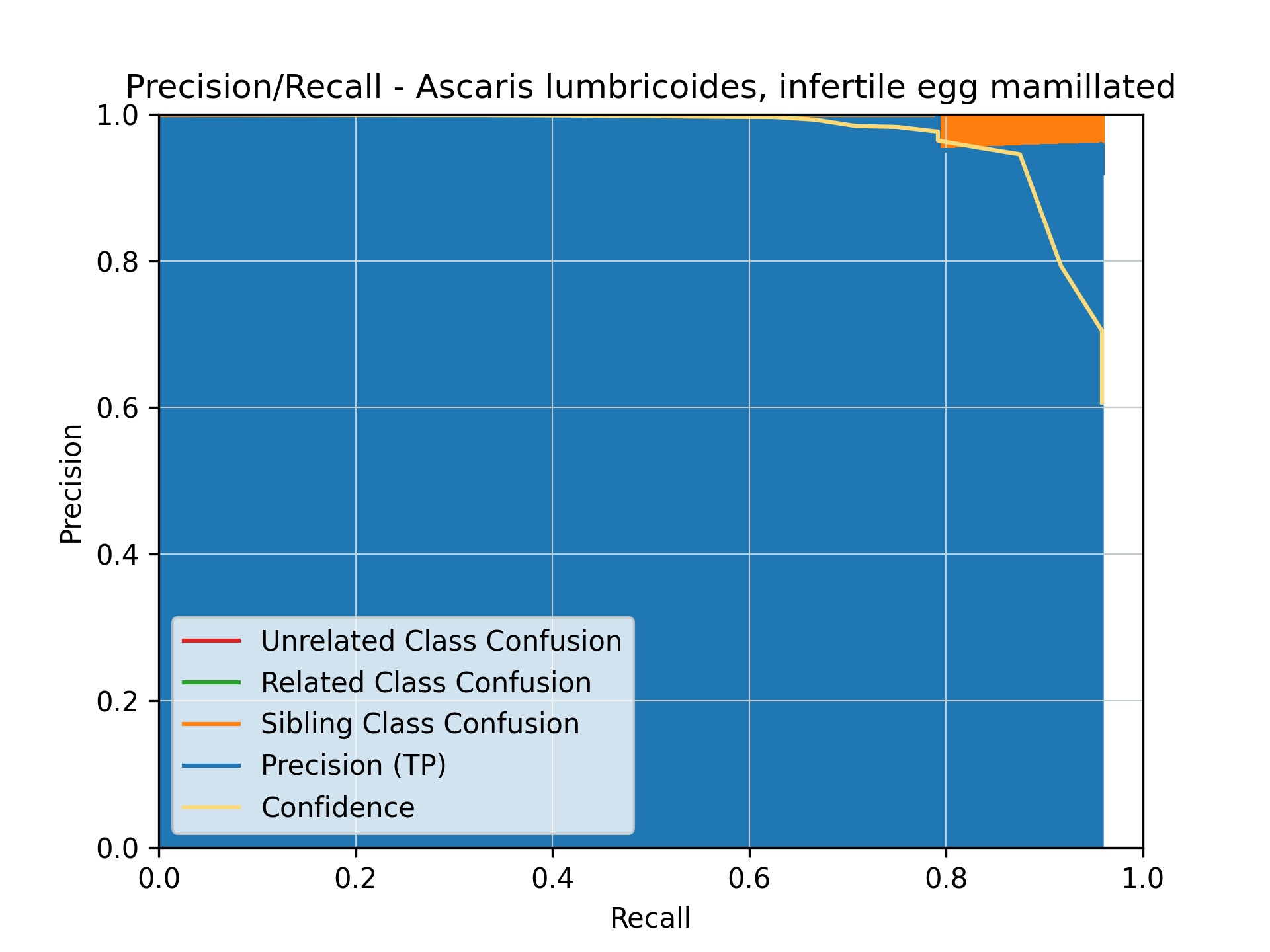

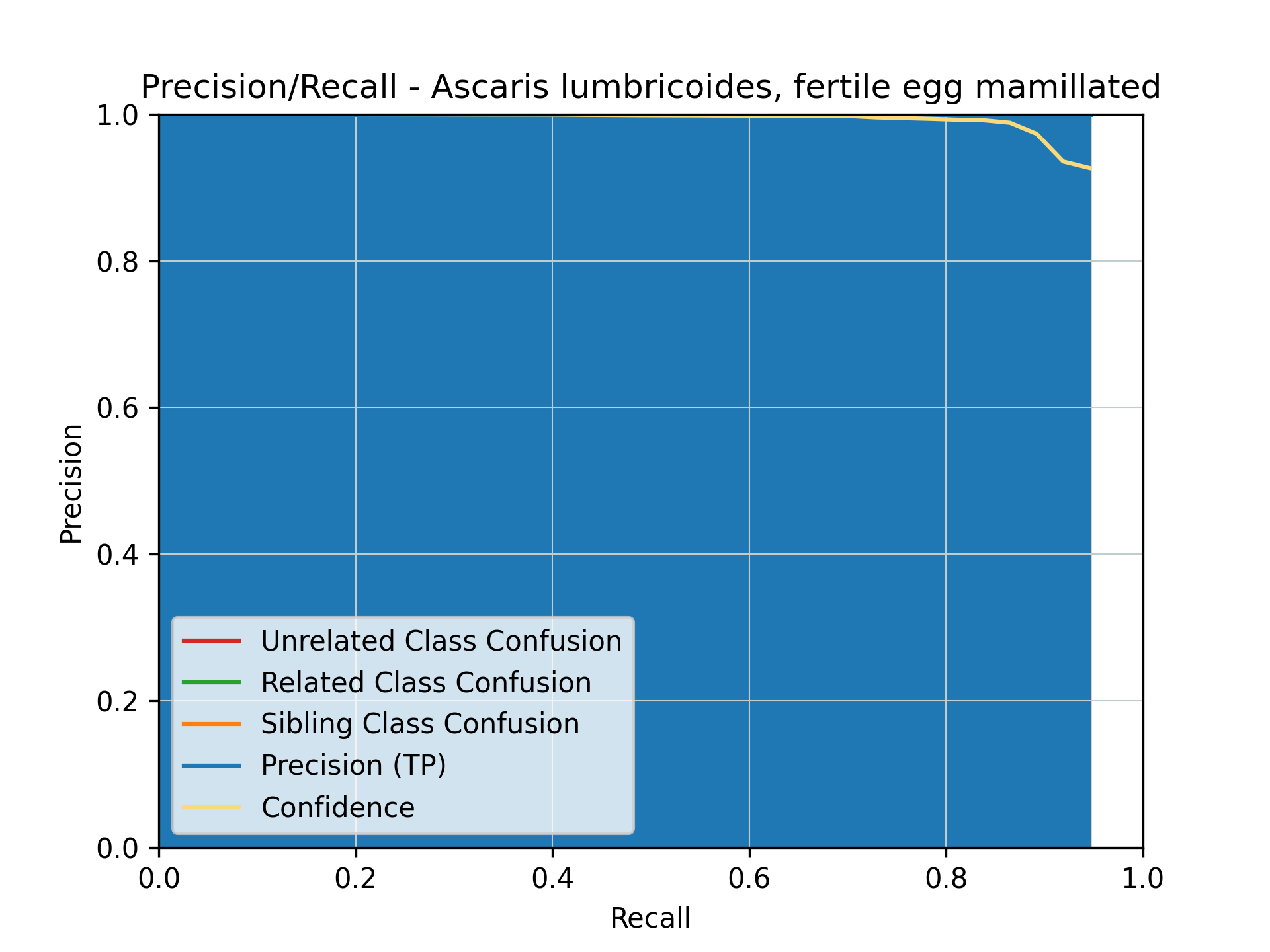

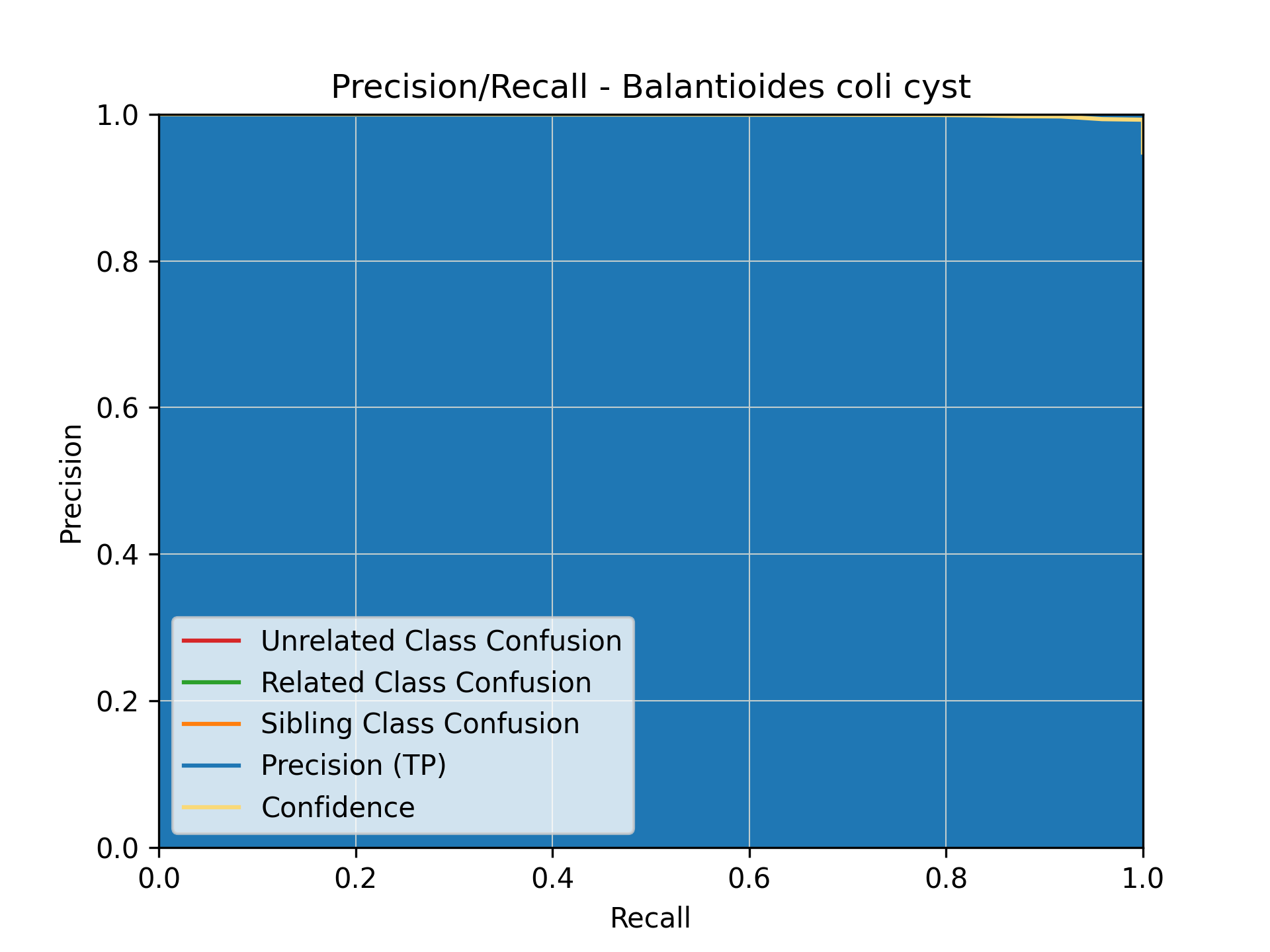

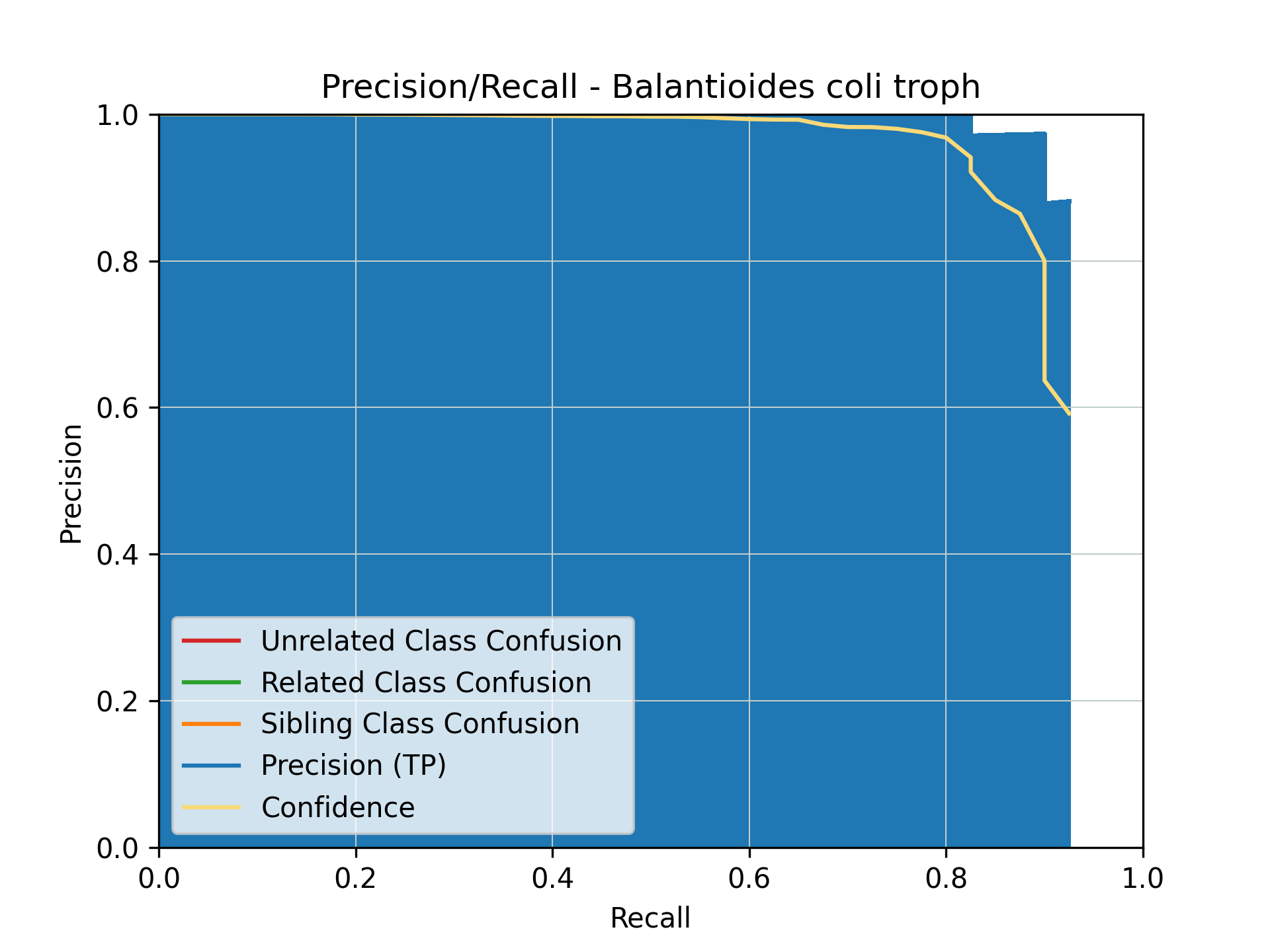

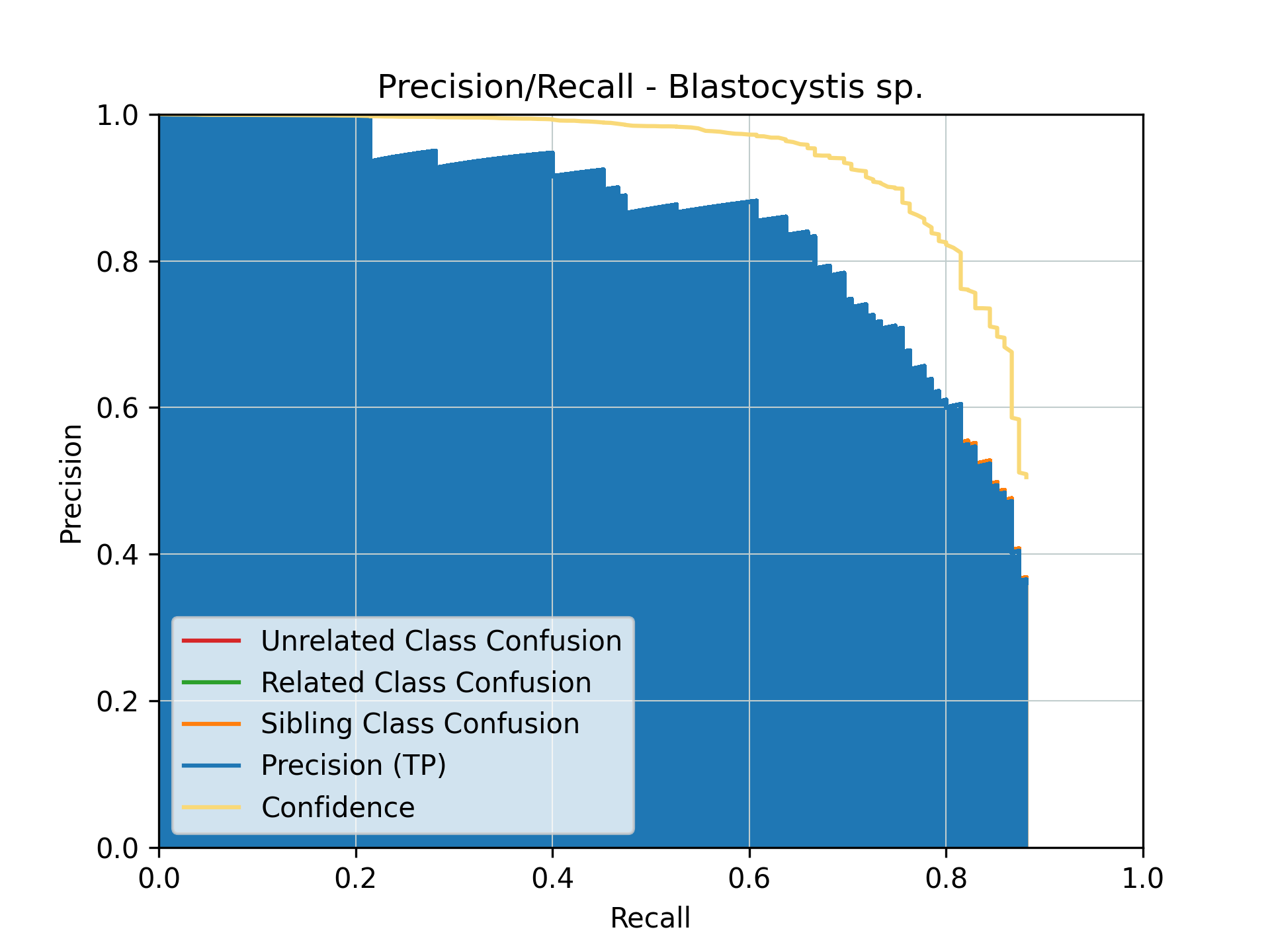

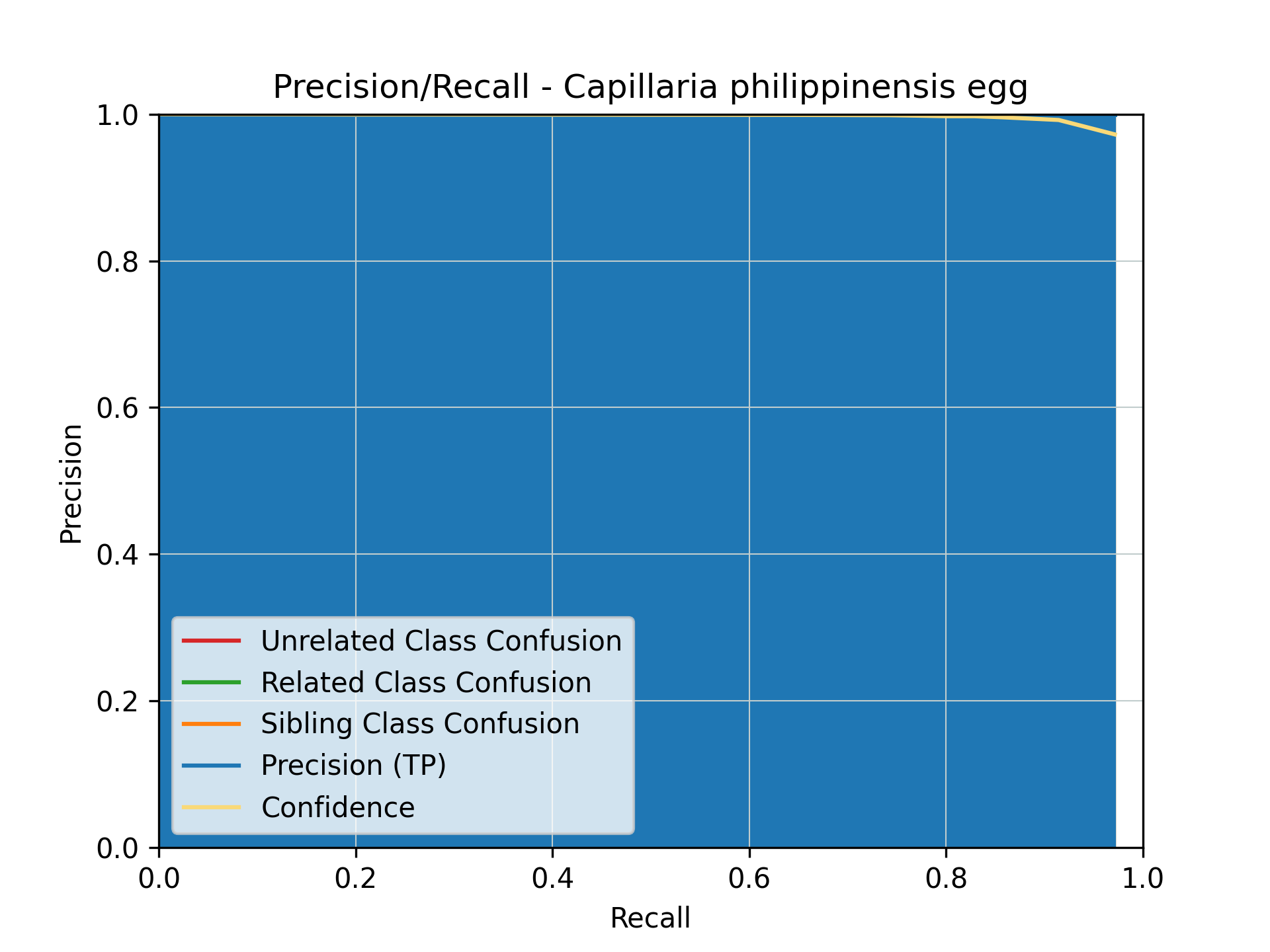

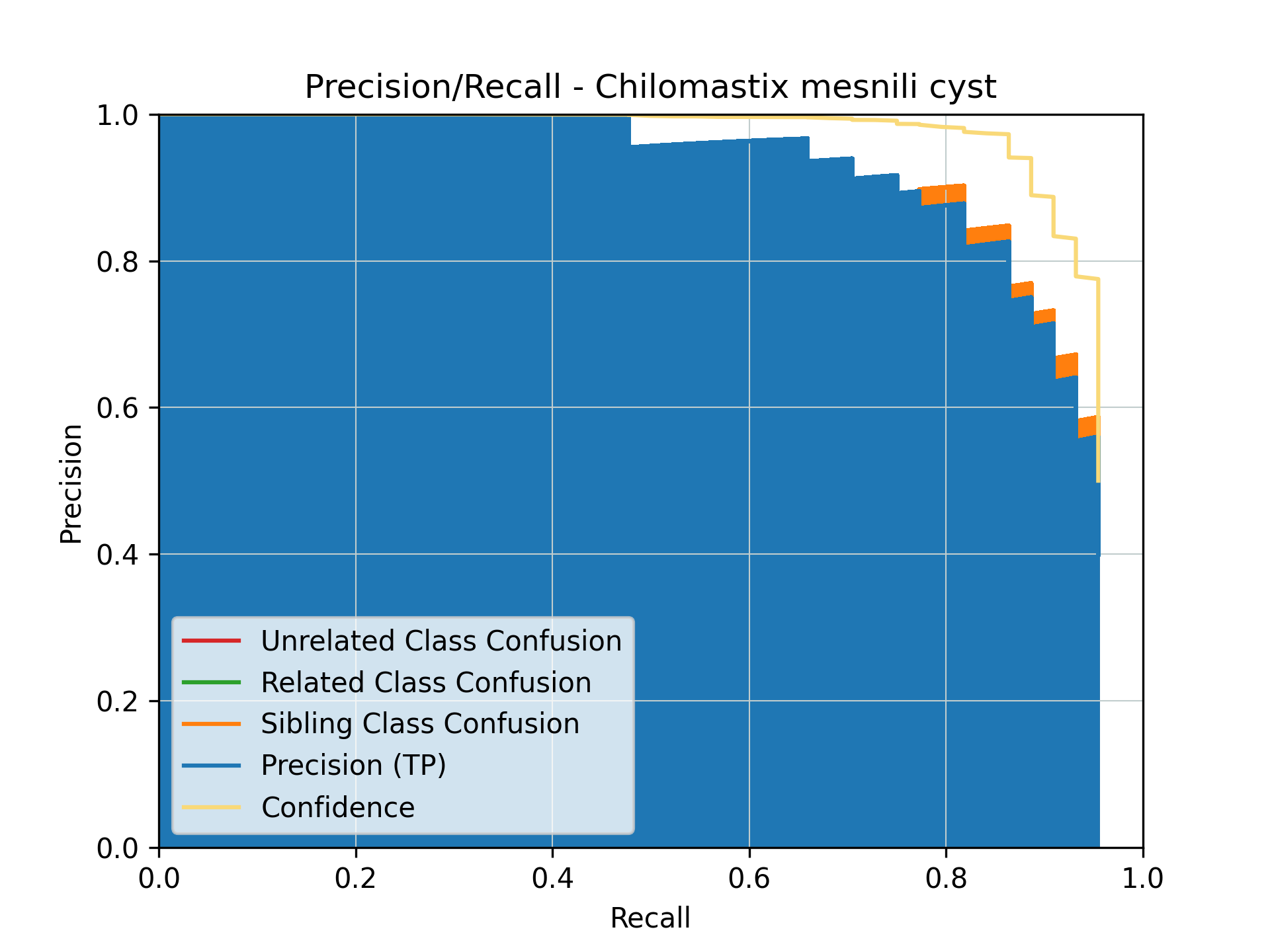

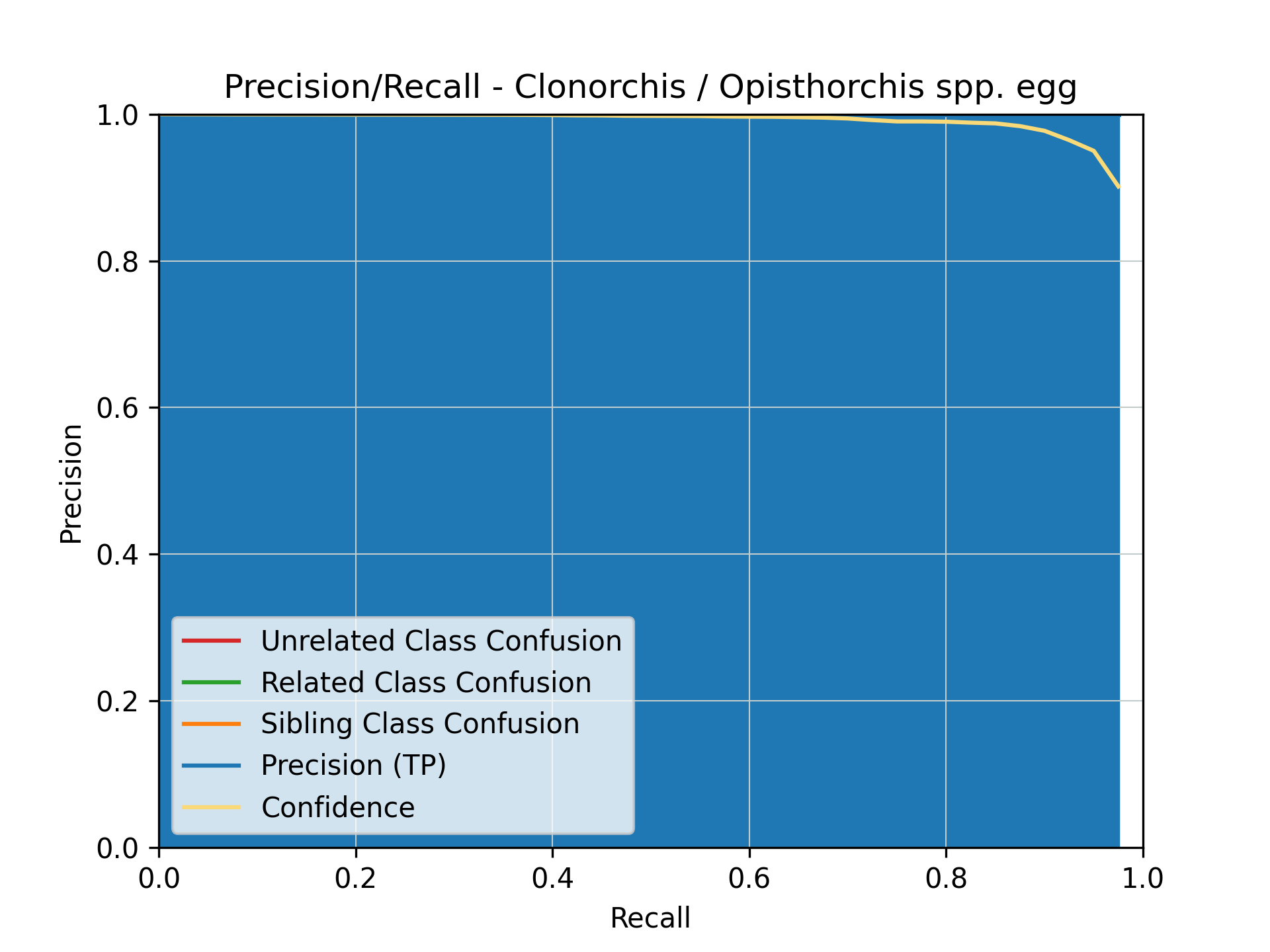

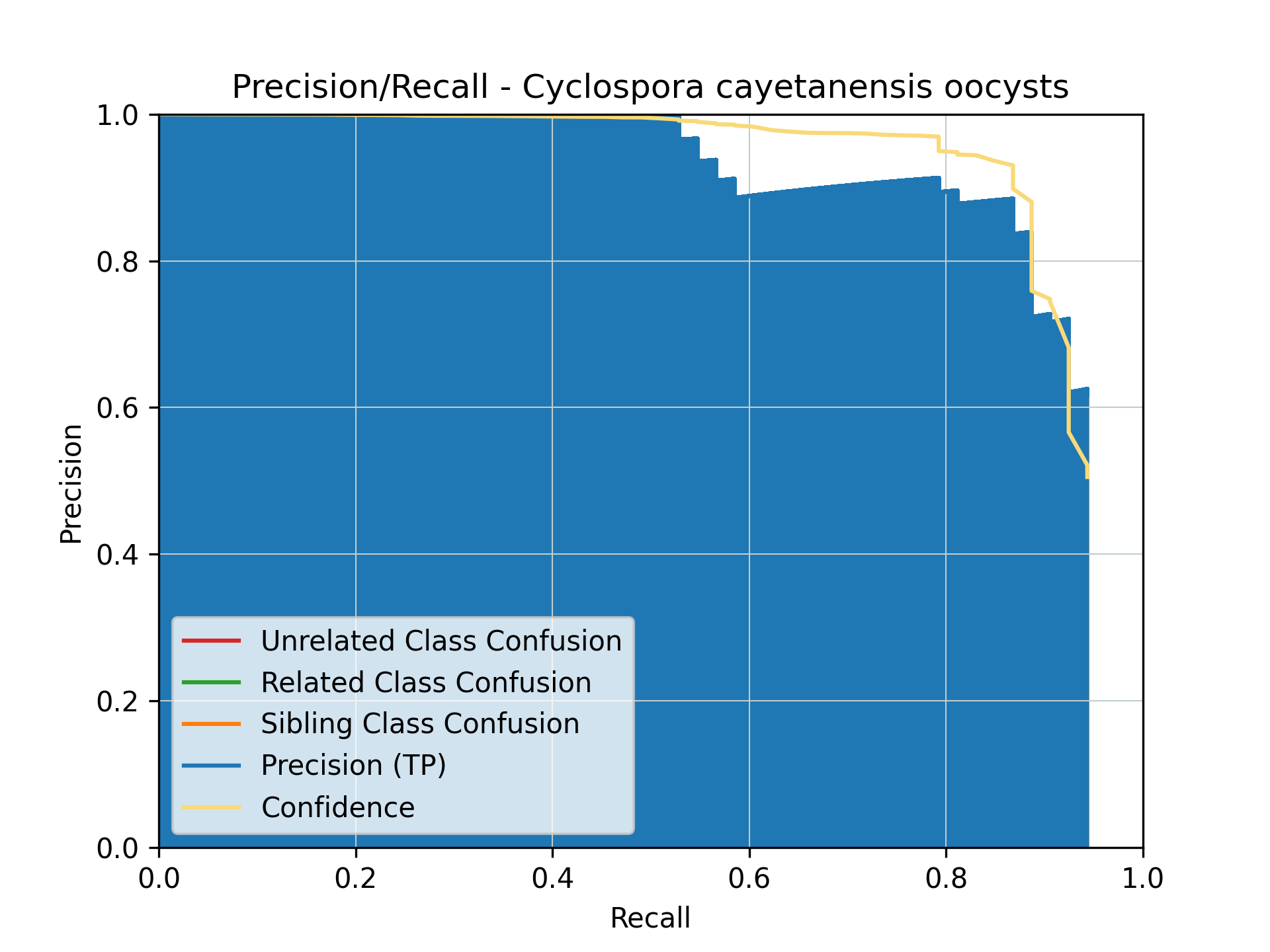

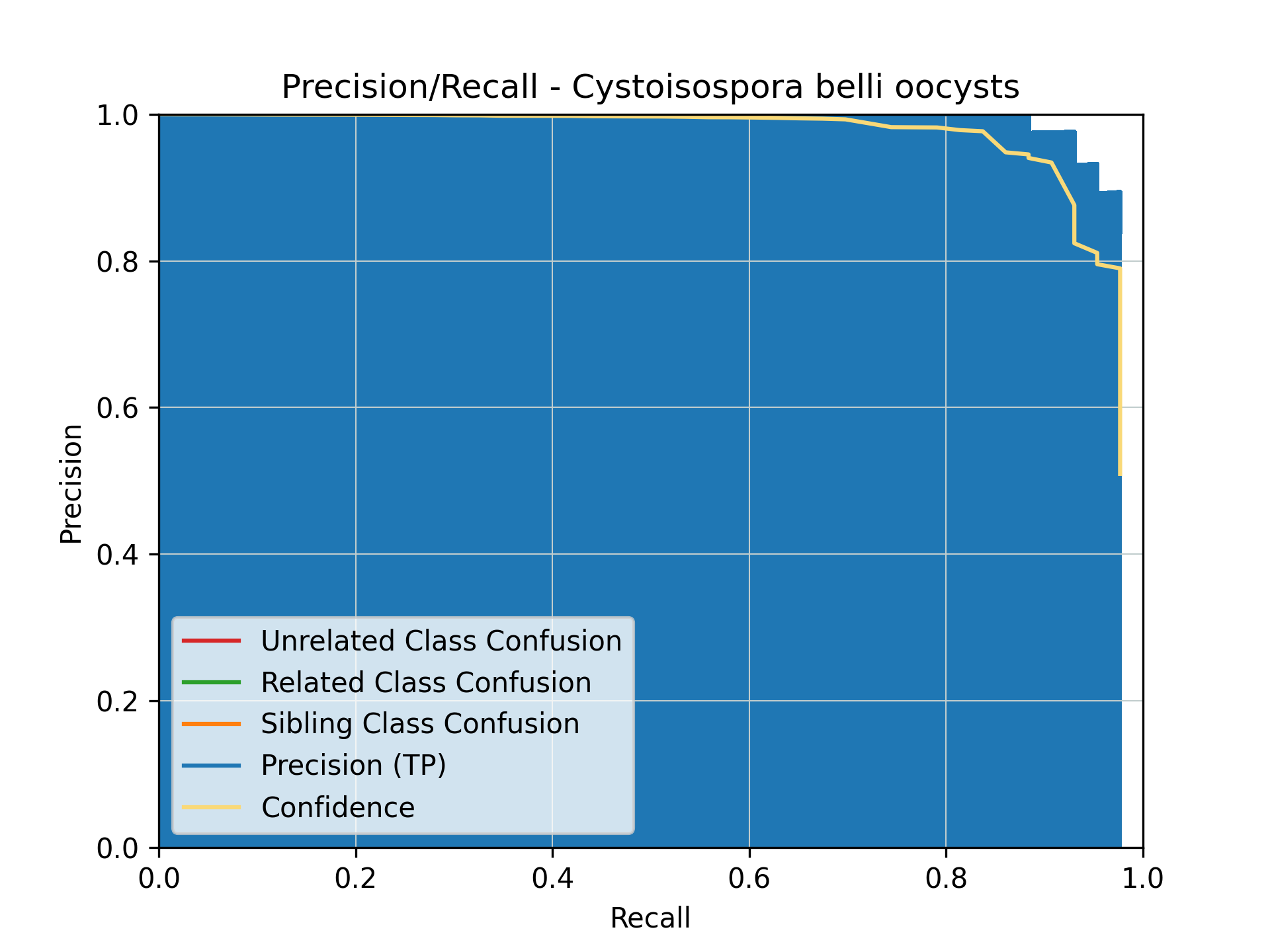

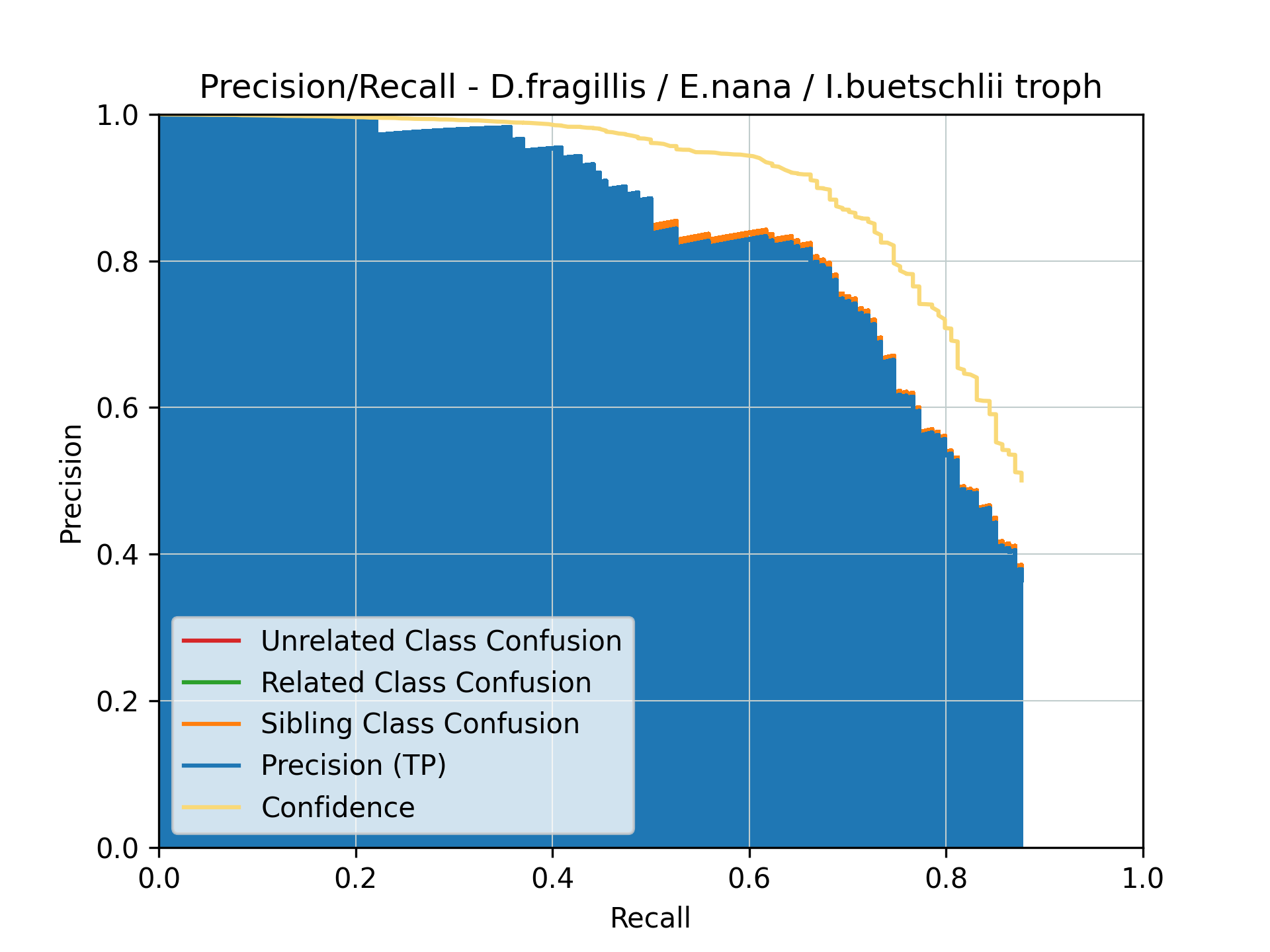

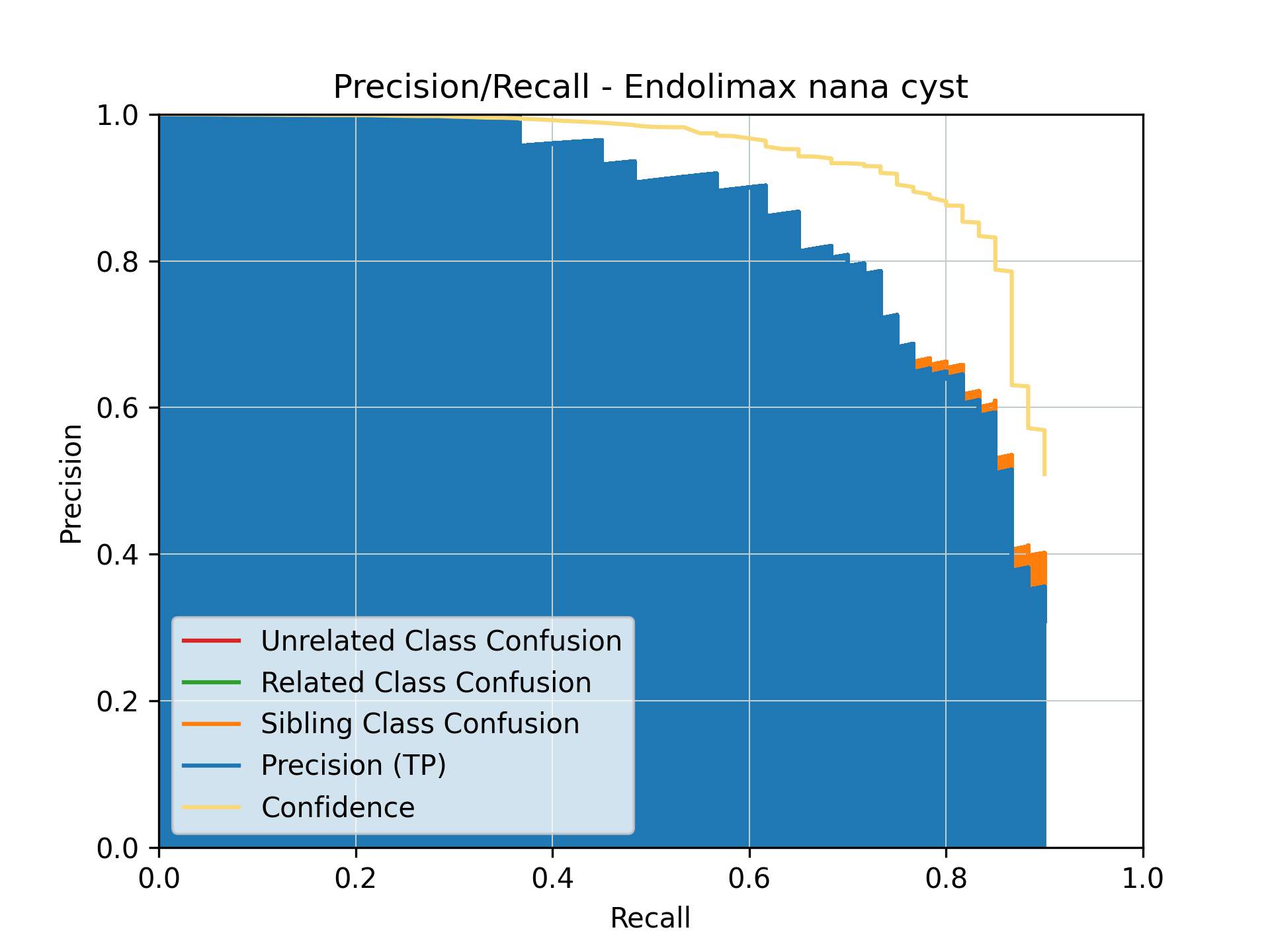

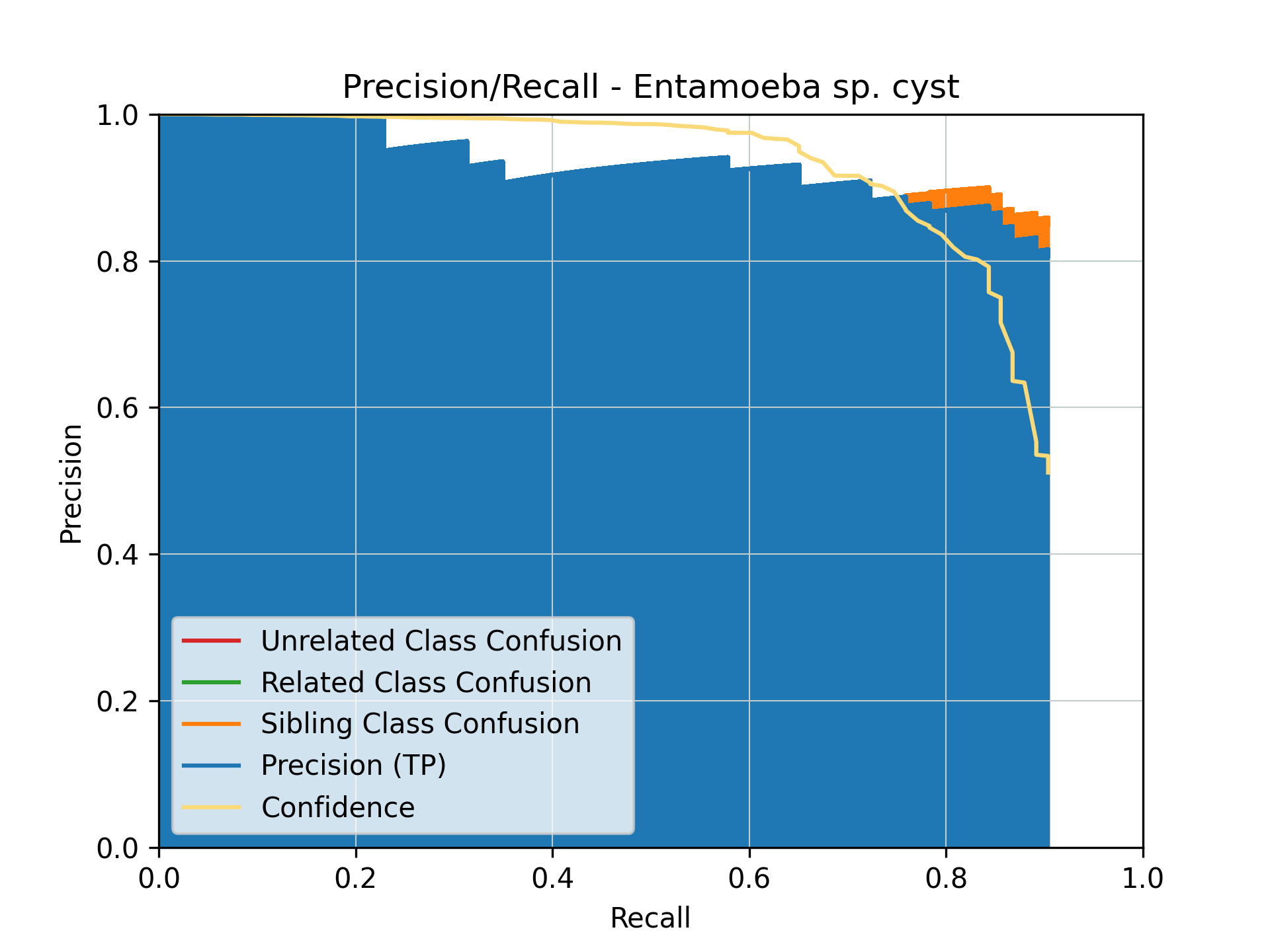

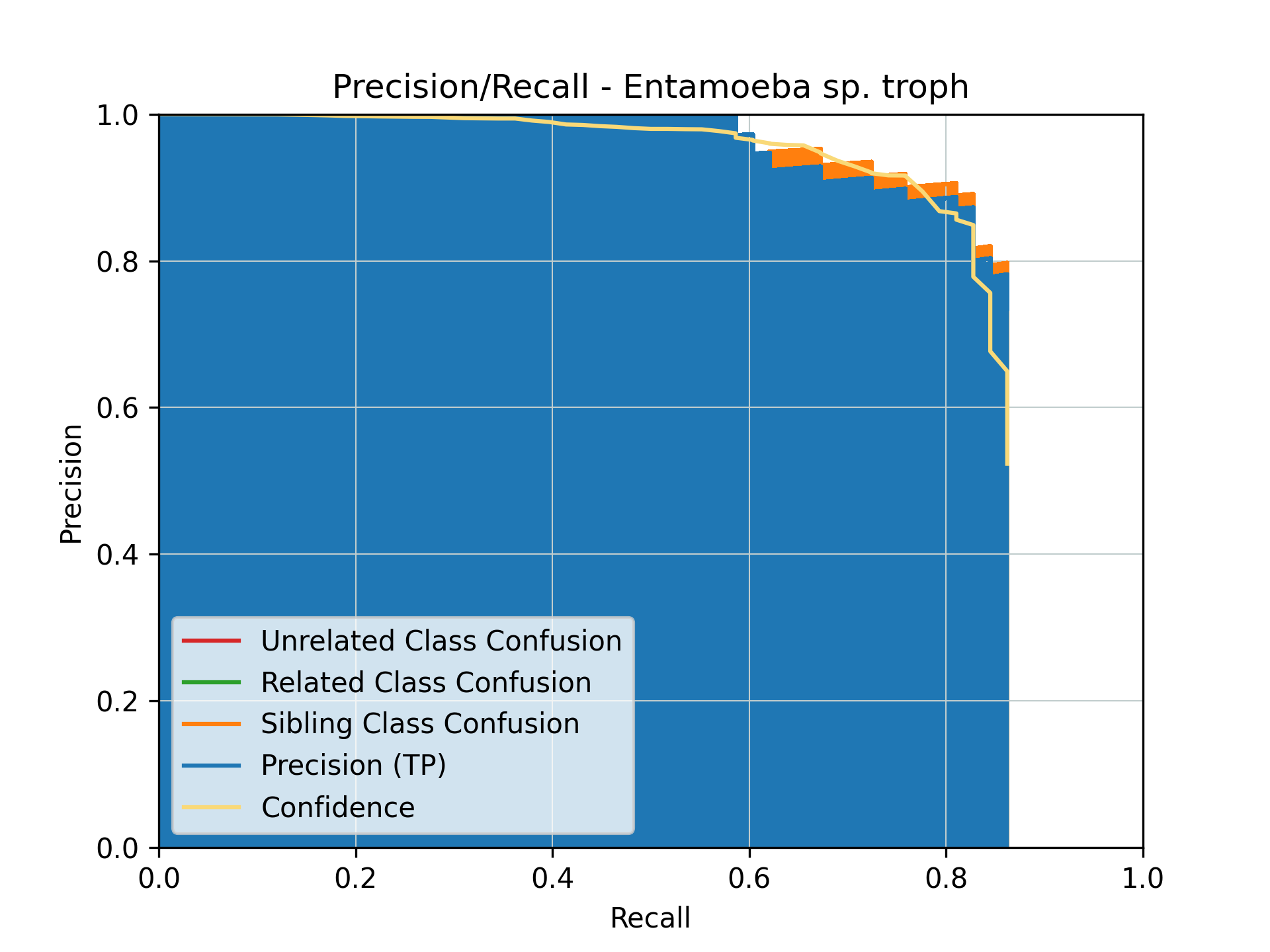

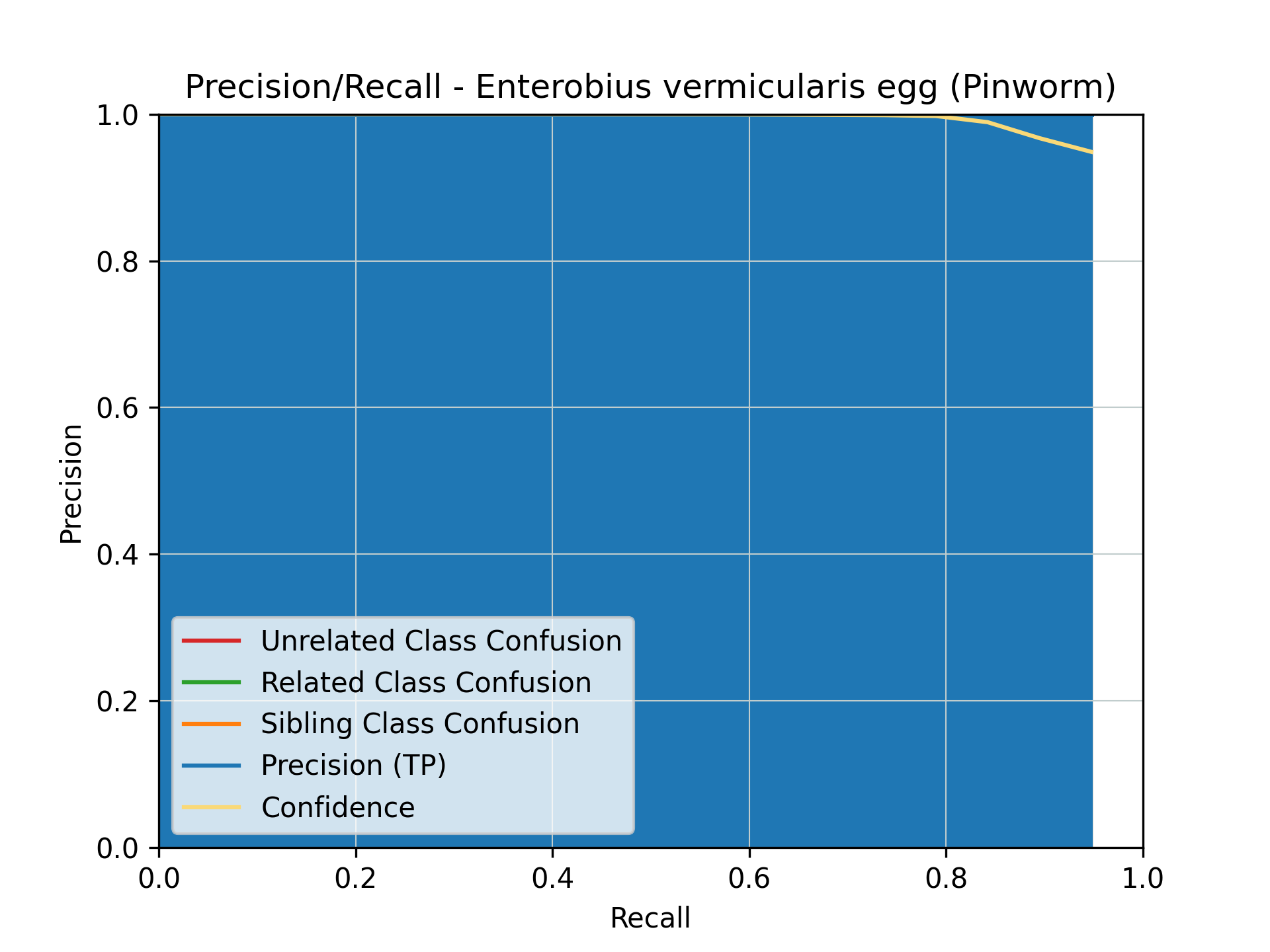

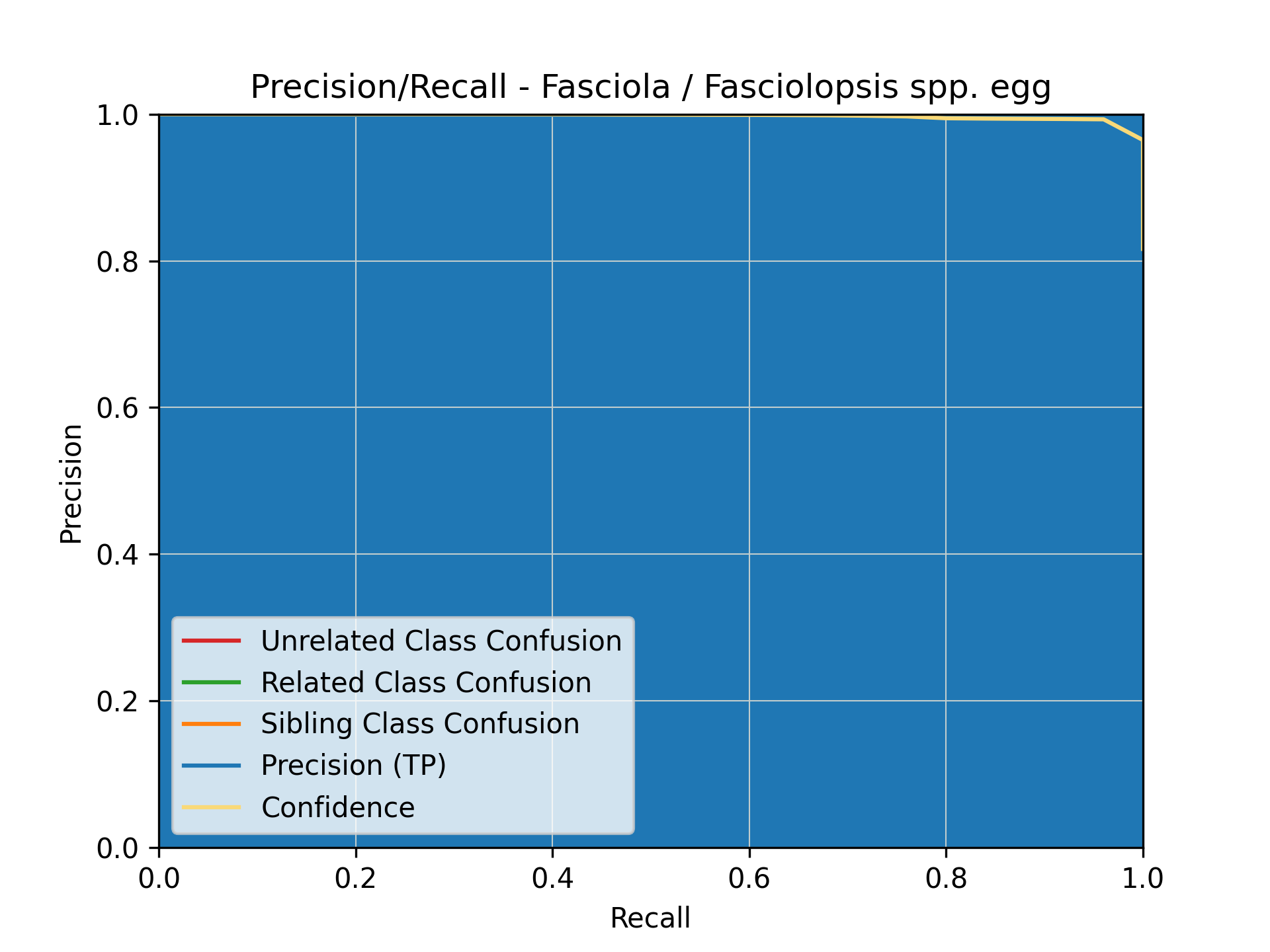

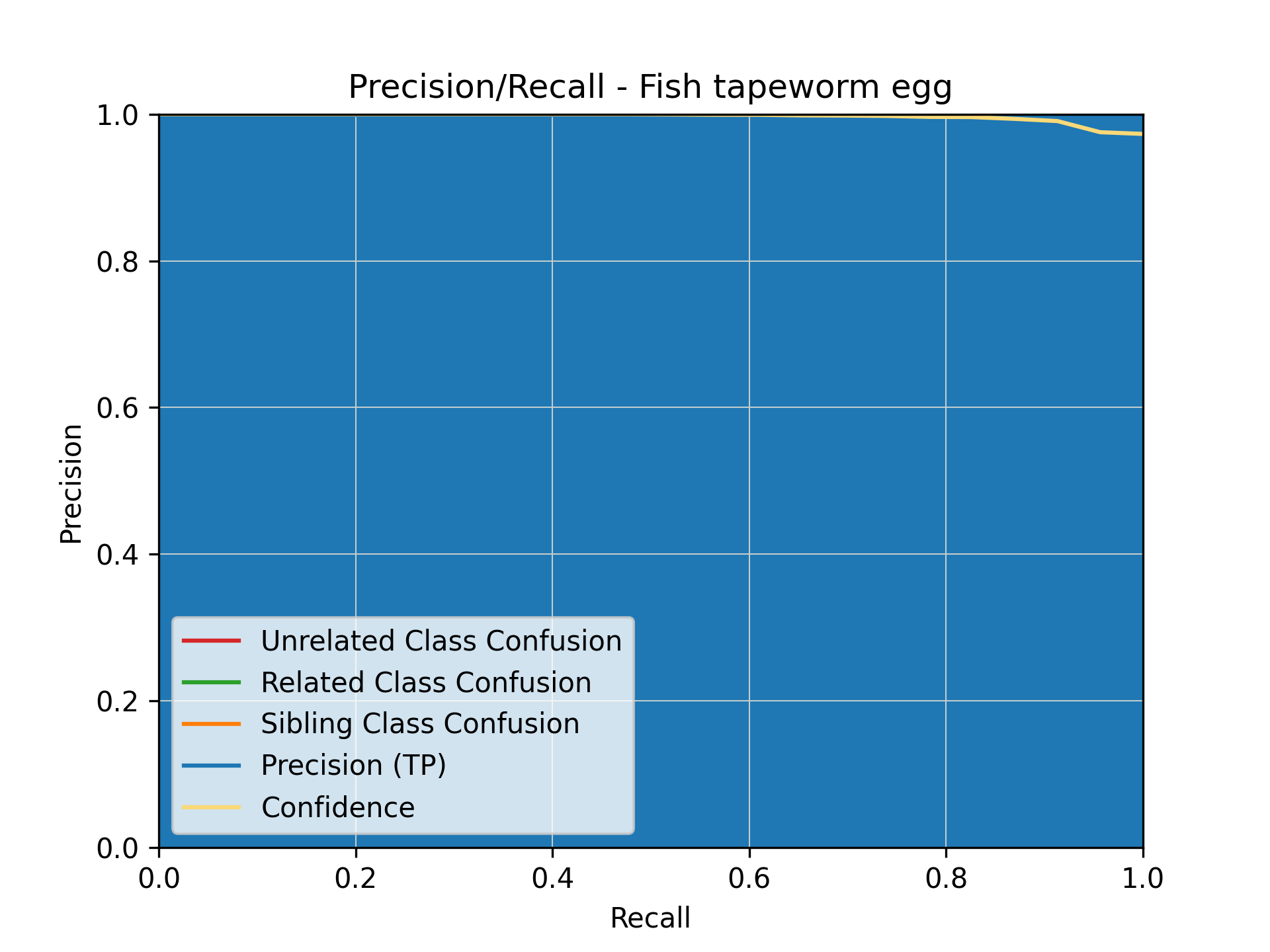

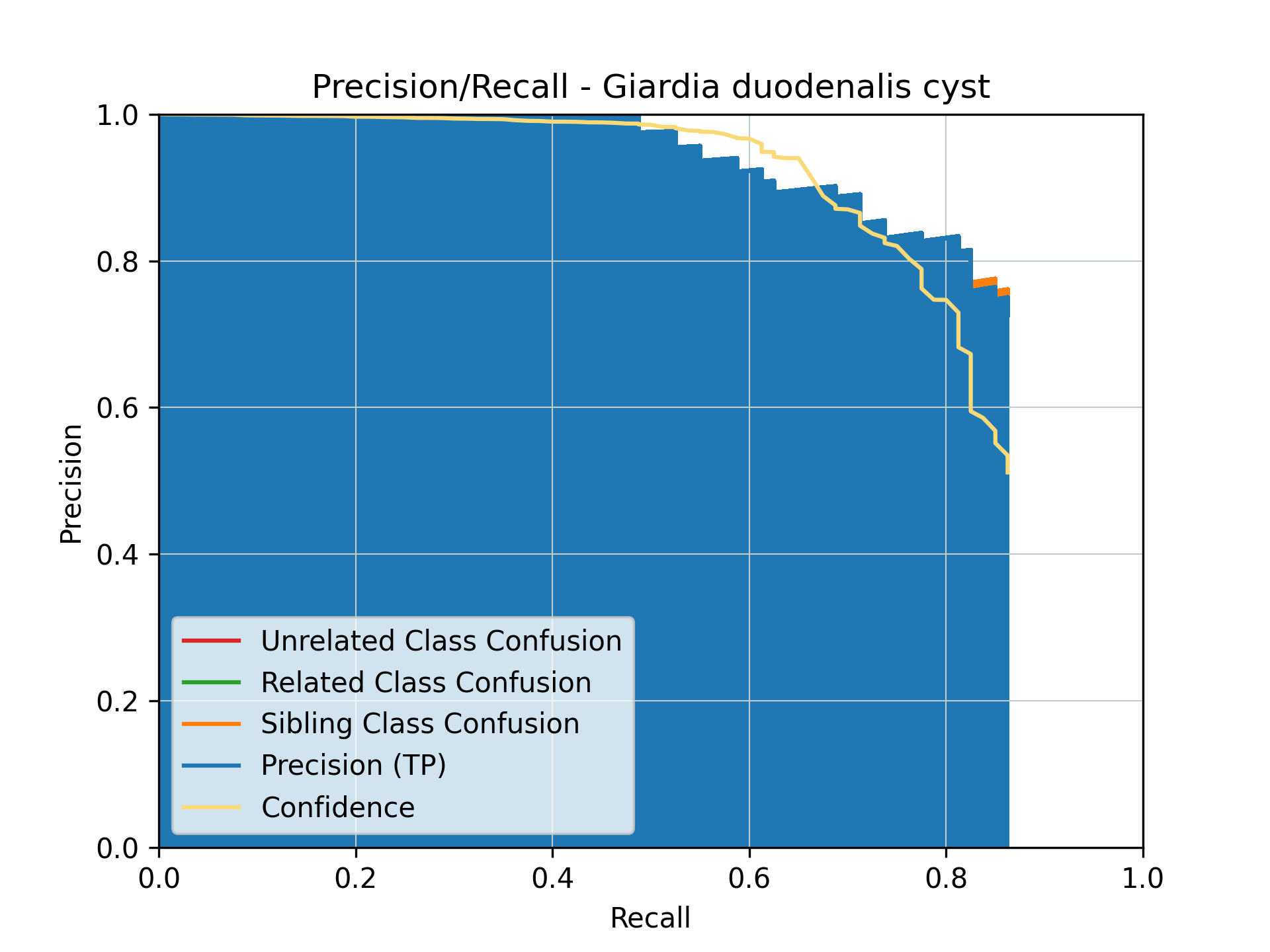

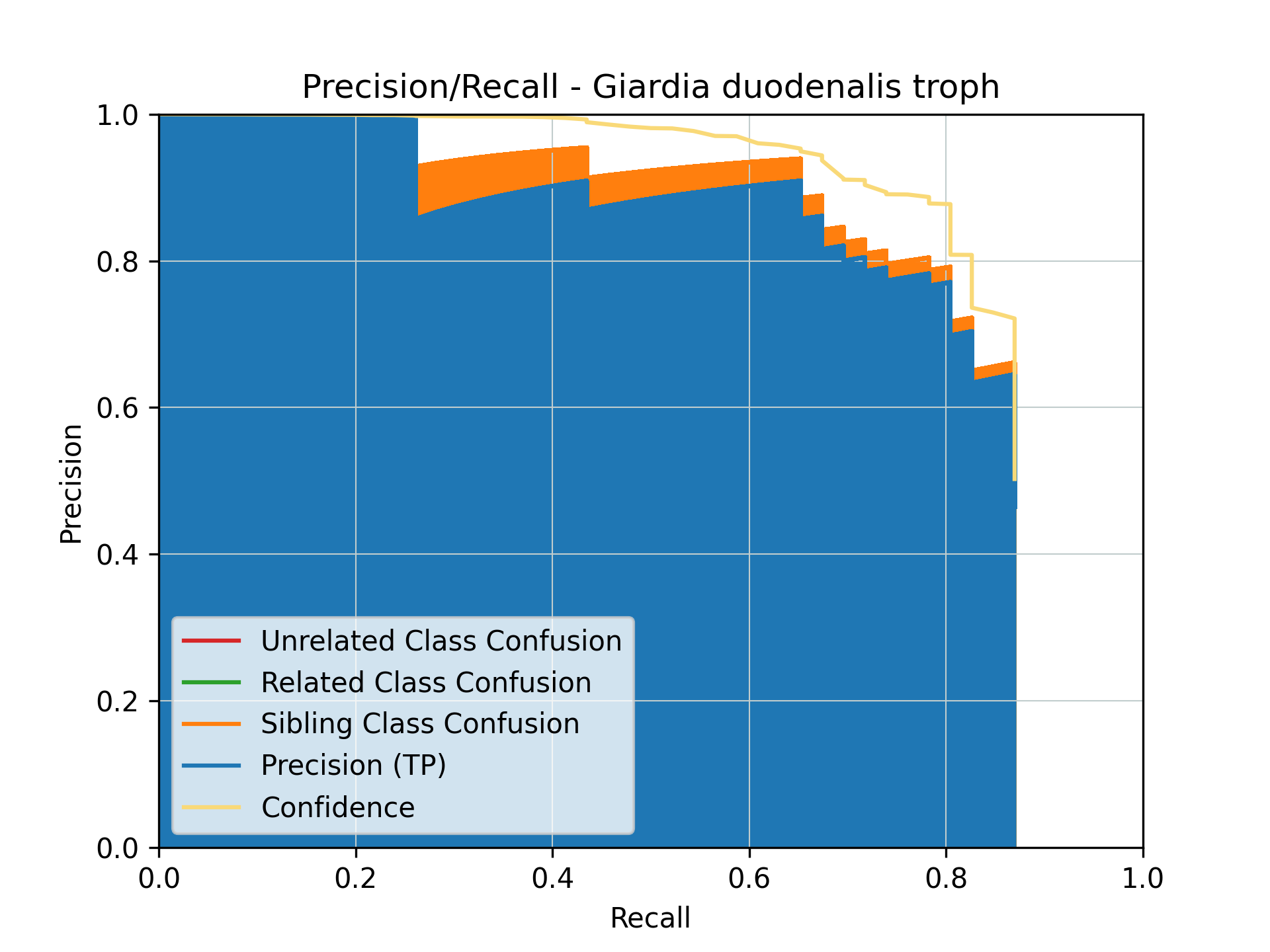

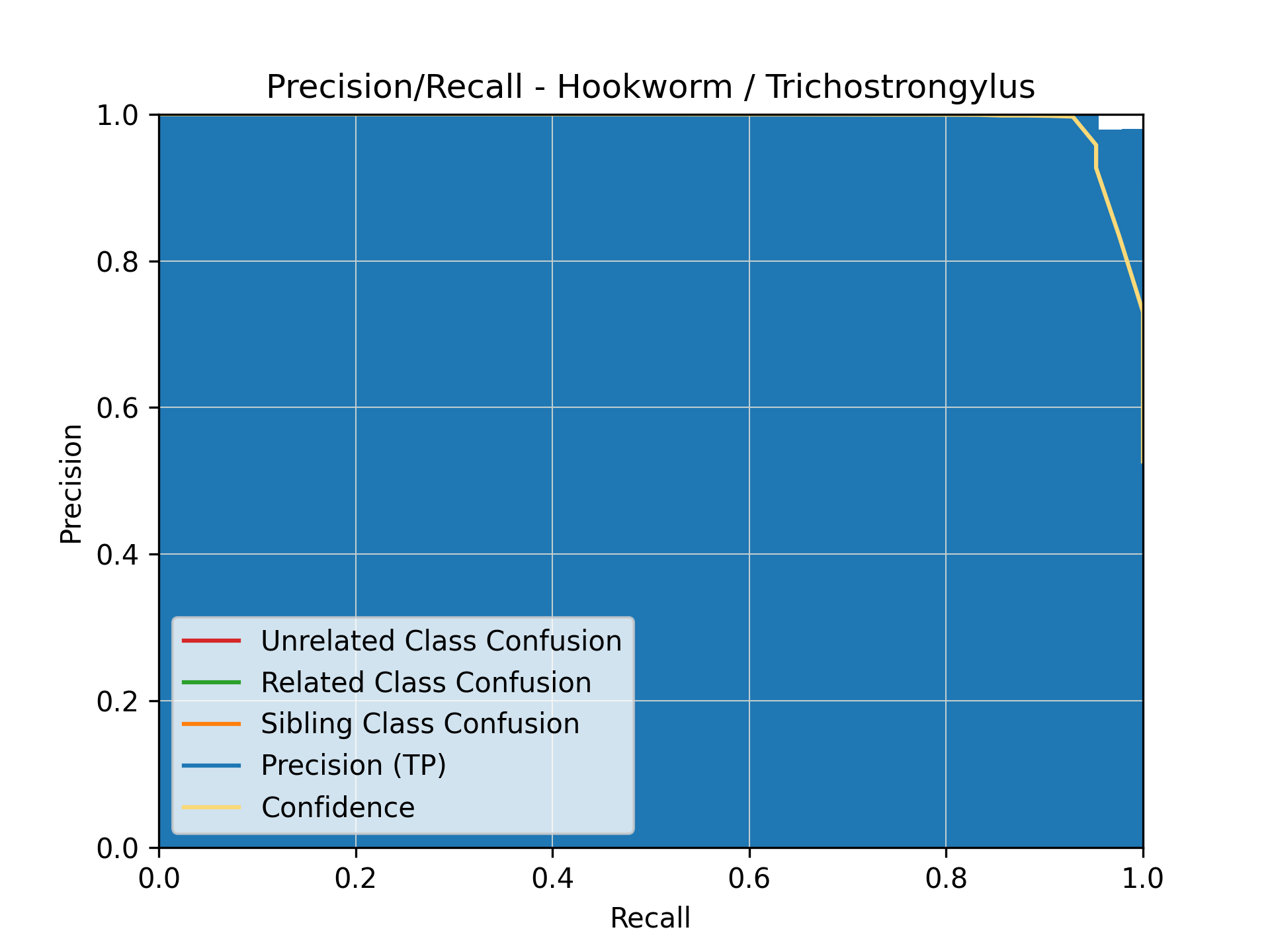

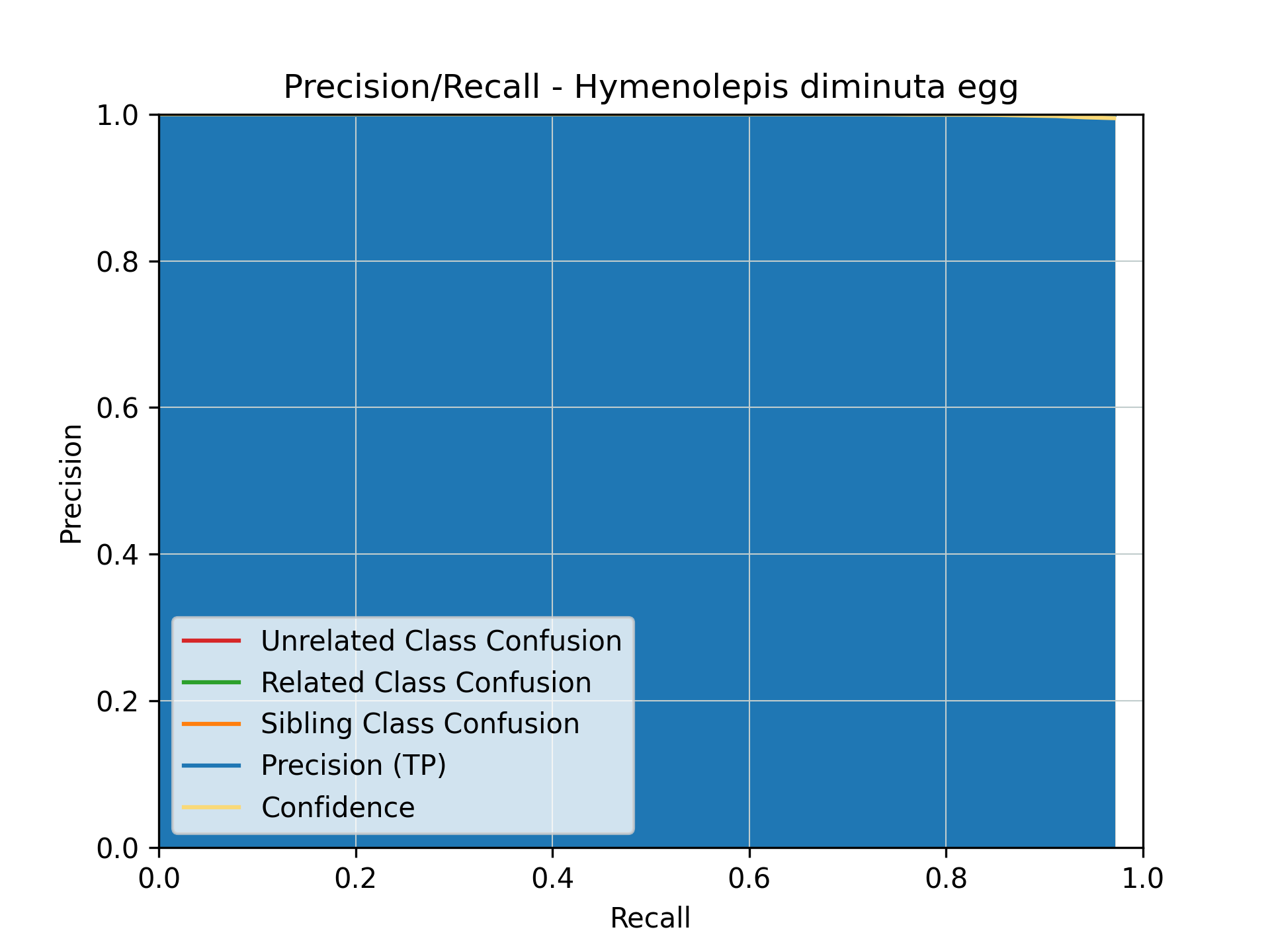

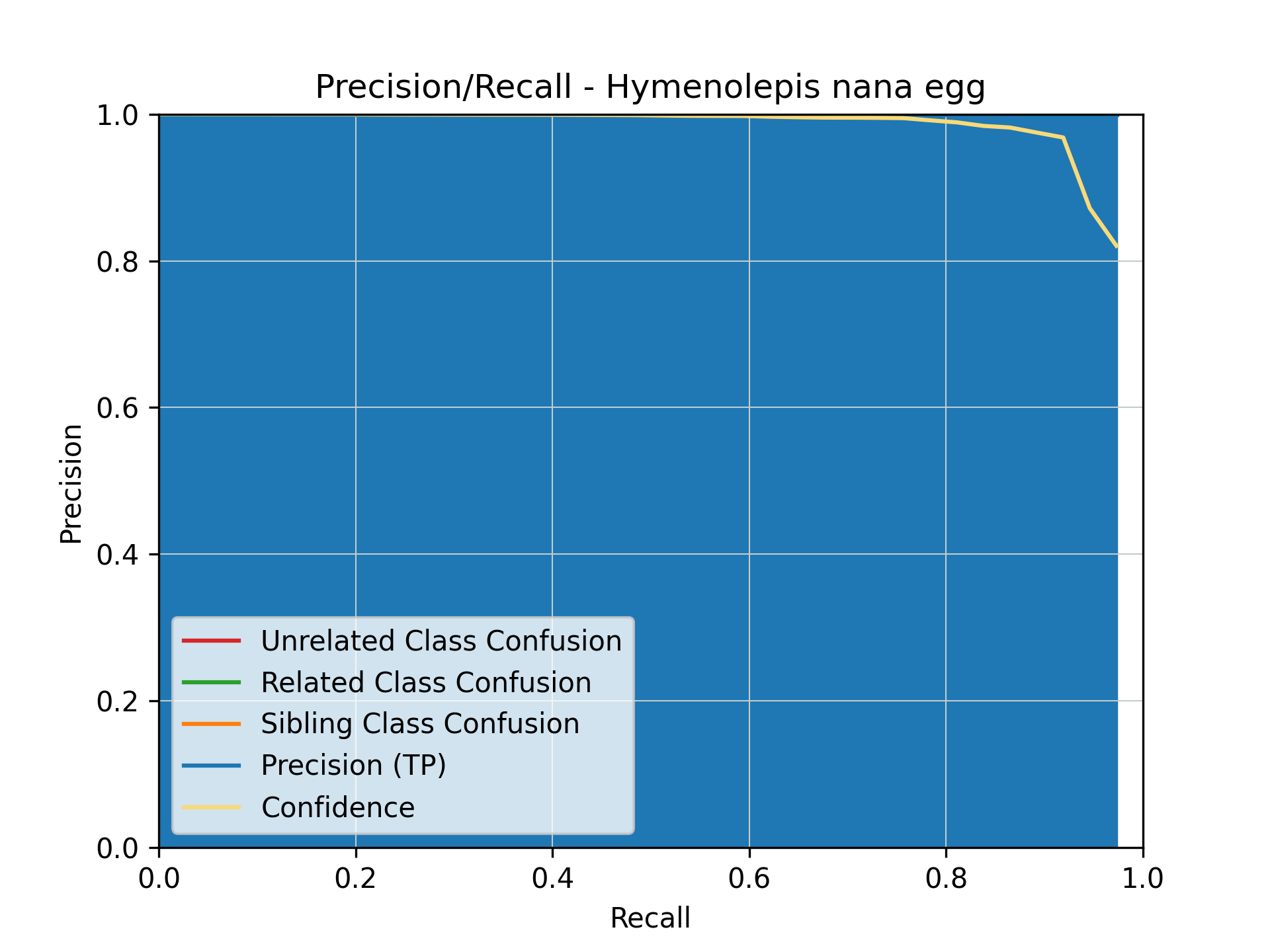

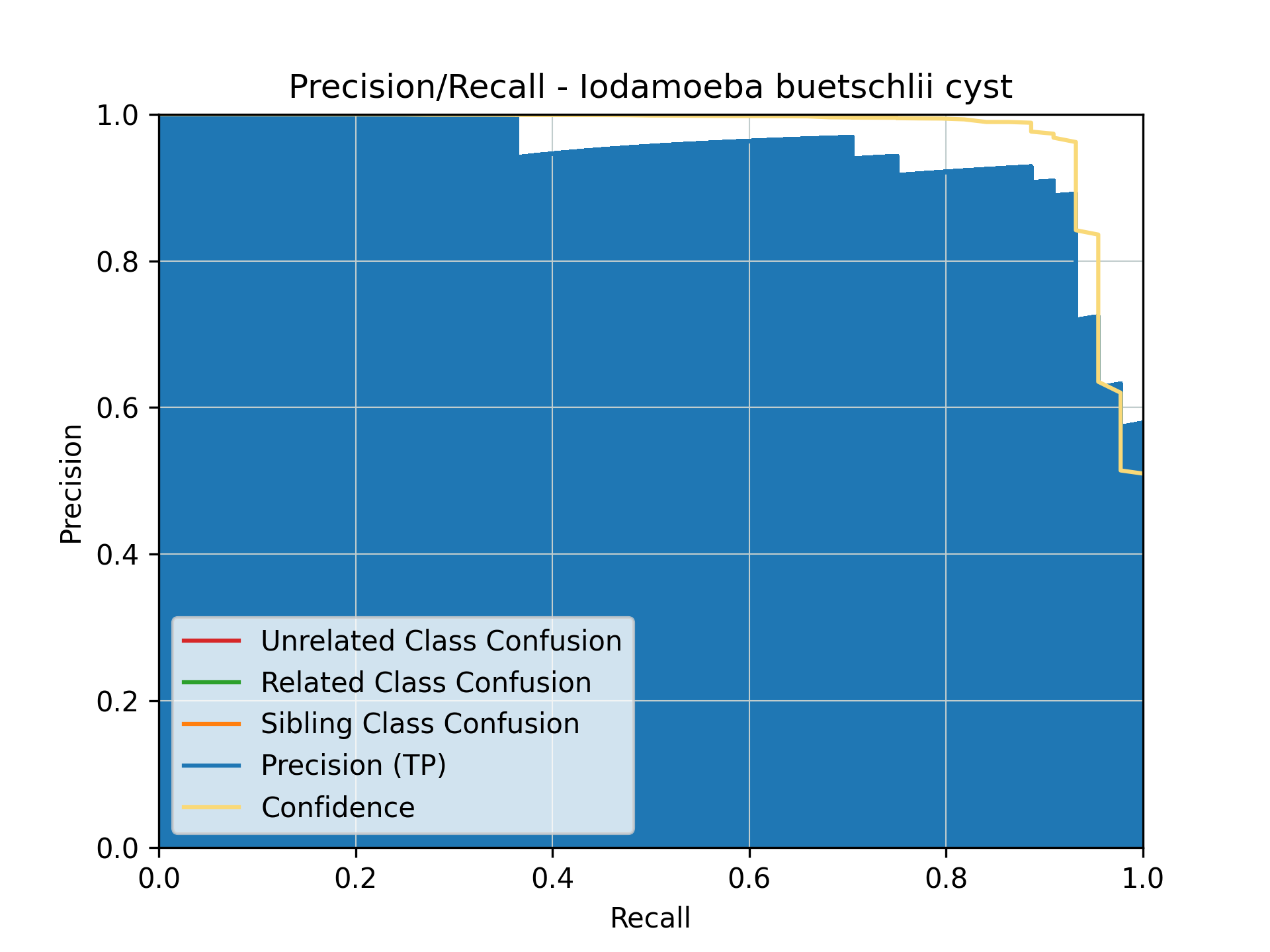

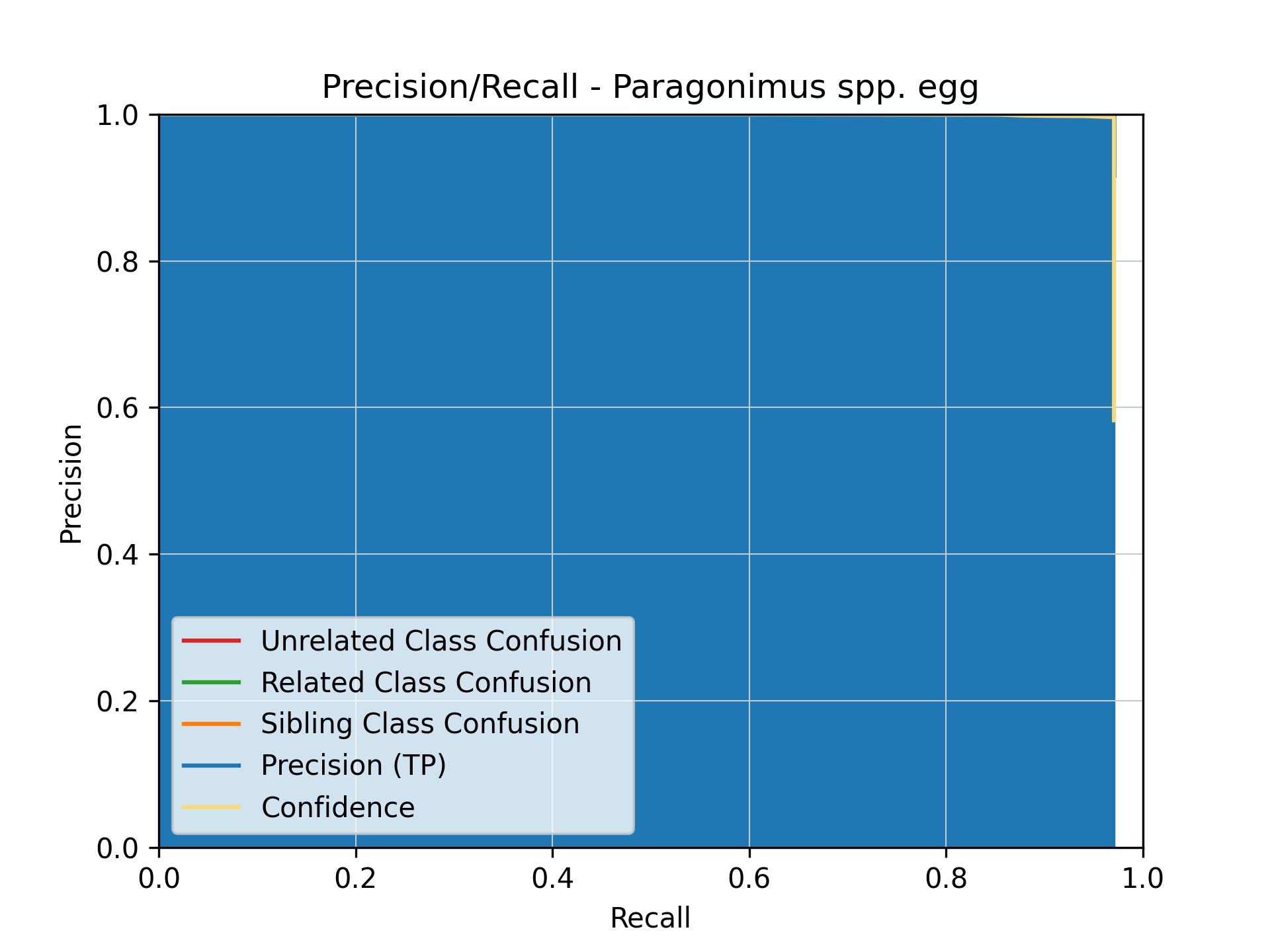

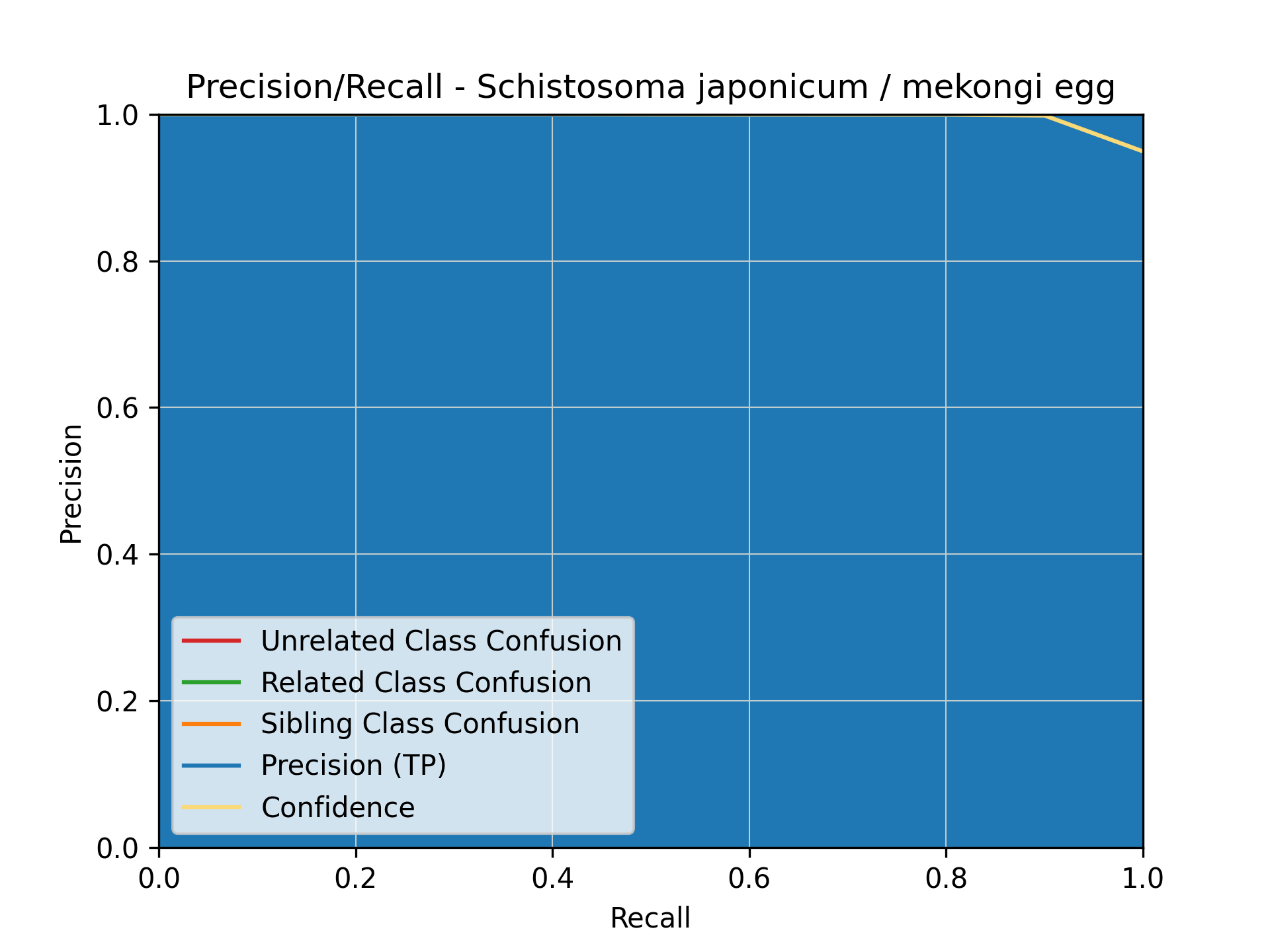

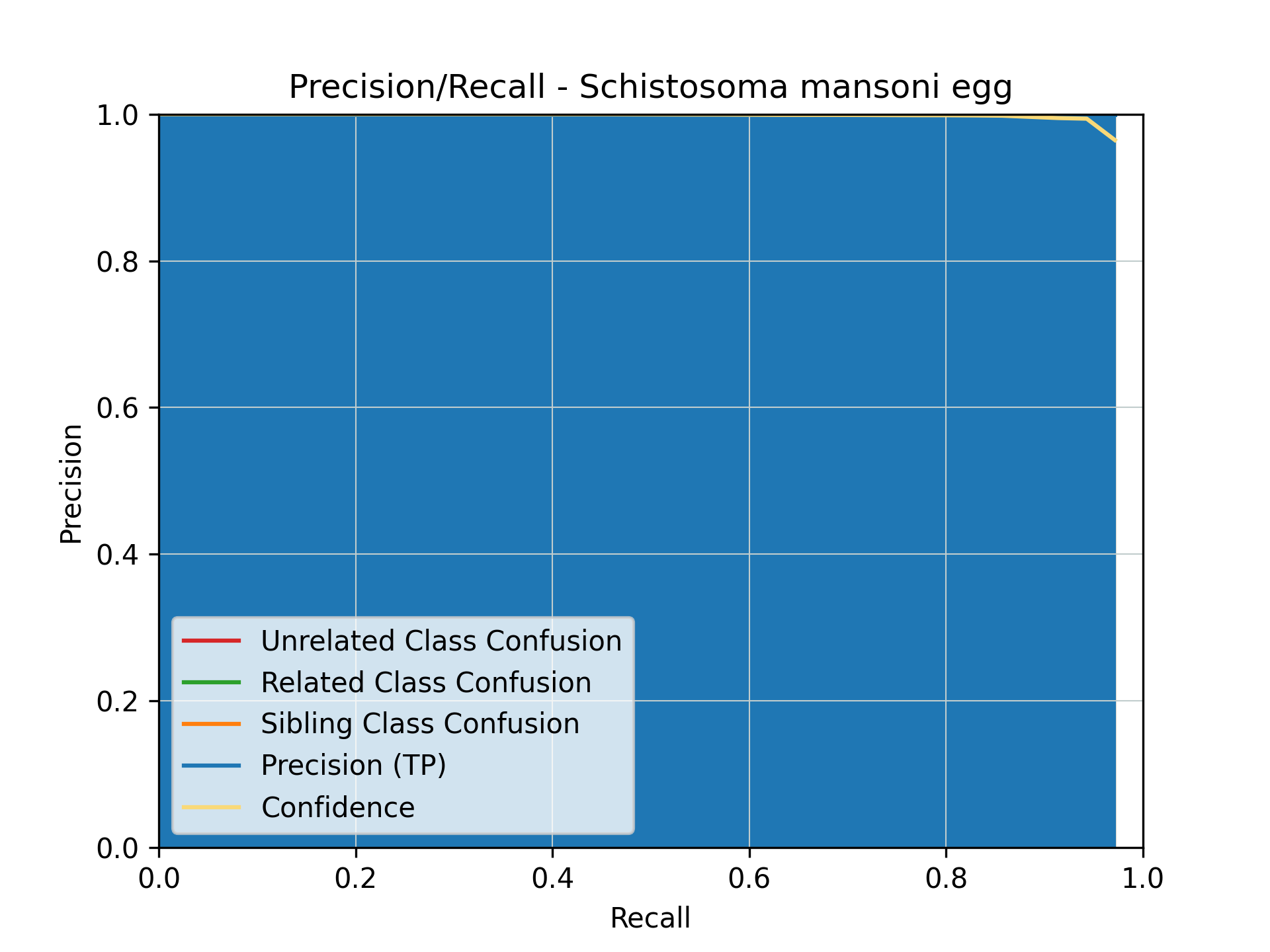

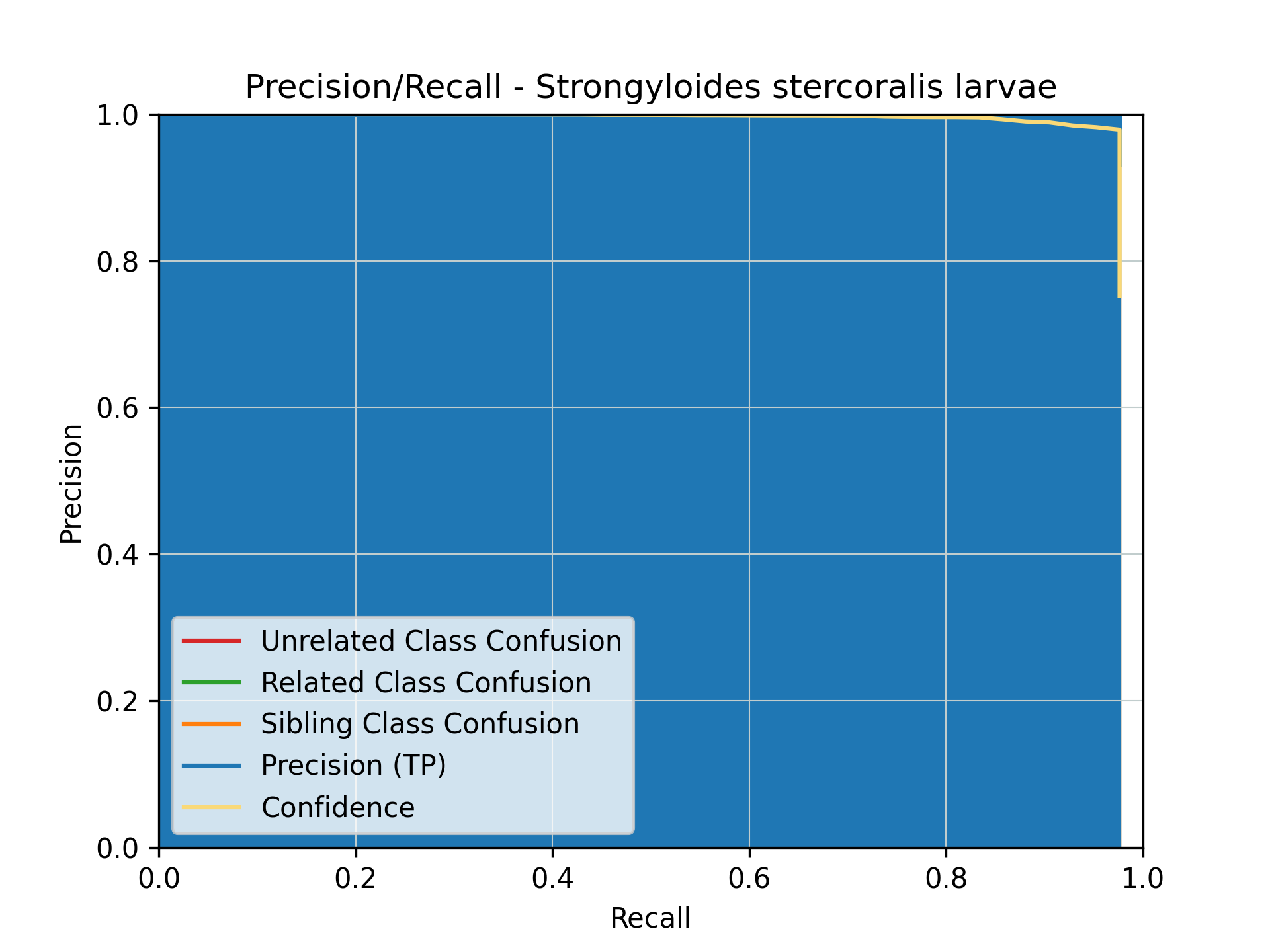

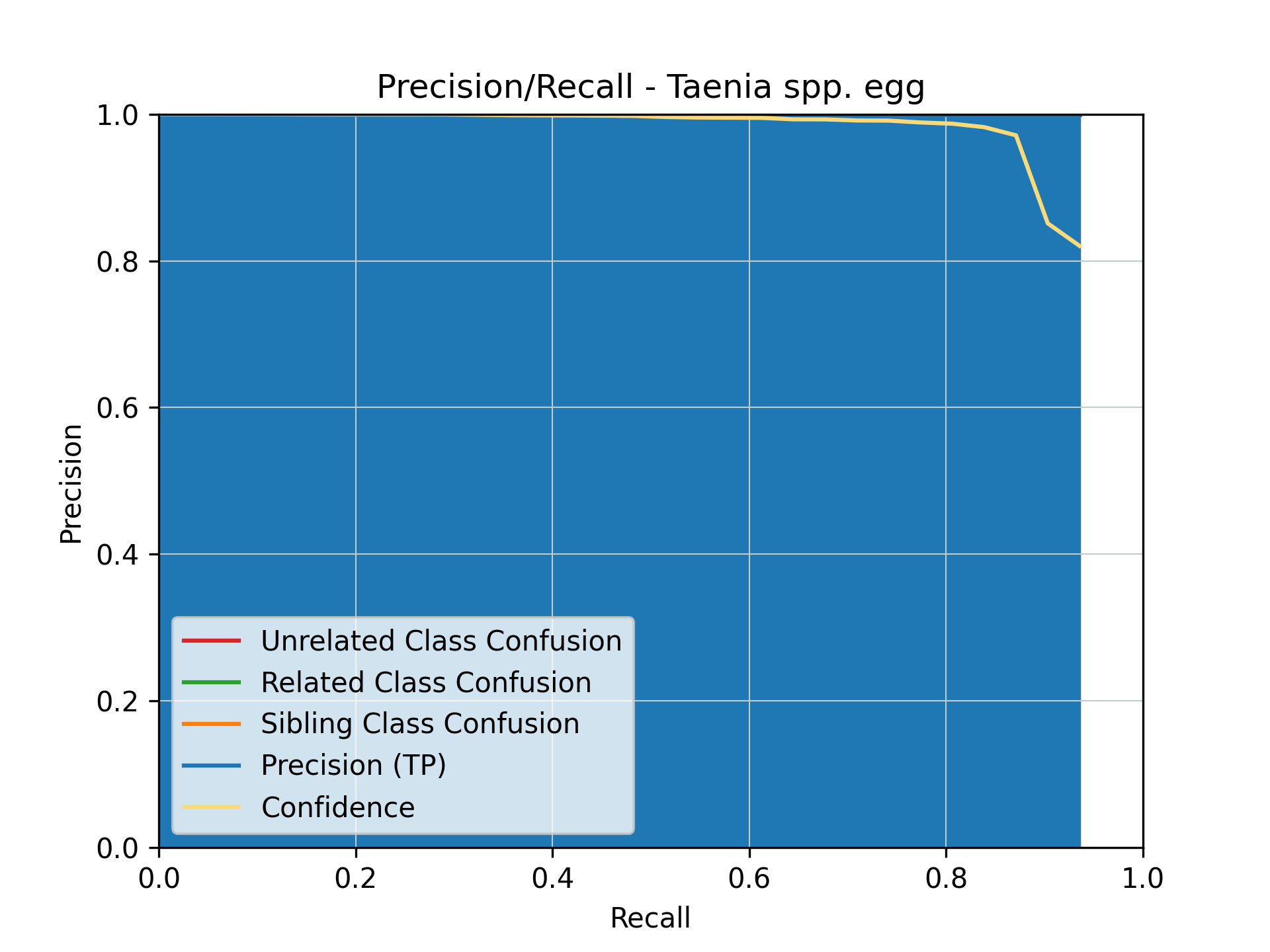

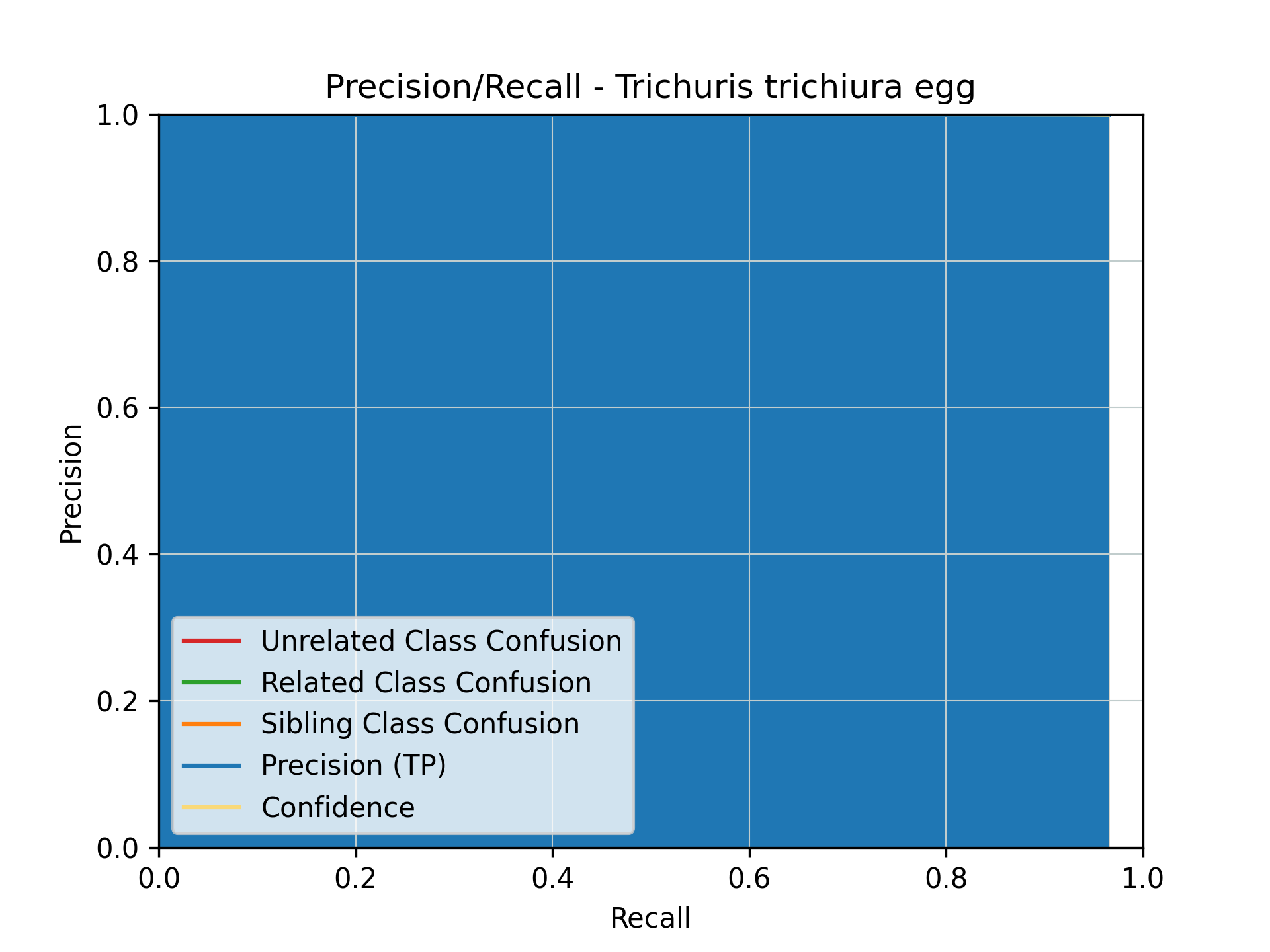


**3. Final thresholds for each class**:

1. *Ascaris lumbricoides*, fertile egg mamillated eggs: 0.90
2. *Ascaris lumbricoides*, infertile egg mamillated eggs: 0.80
3. *Balantioides coli* cyst: 0.90
4. *Balantioides coli* trophozoites: 0.90
5. *Blastocystis* spp.: 0.93
6. *Paracapillaria philippinensis* egg: 0.80
7. *Chilomastix mesnili* cyst: 0.95
8. *Chilomastix mesnili* trophozoites: 0.95
9. *Clonorchis*/*Opisthorchis* spp. eggs 0.95
10. *Cyclospora* spp. oocysts: 0.85
11. *Cystoisospora belli* oocysts: 0.90
12. Misc. small protozoans: 0.90
13. *Endolimax nana* cysts 0.94
14. *Entamoeba* sp. (non-*hartmanni*) cysts: 0.80
15. *Entamoeba* sp. (non-*hartmanni*) trophozoites: 0.80
16. *Enterobius vermicularis* eggs: 0.90
17. *Fasciola*/*Fasciolopsis* spp. eggs 0.85
18. Fish tapeworm eggs: 0.90
19. *Giardia duodenalis* cysts: 0.80
20. *Giardia duodenalis* trophozoites: 0.92
21. Hookworm/Trichostrongylus eggs: 0.85
22. *Hymenolepis diminuta* eggs: 0.90
23. *Rodentolepis nana* eggs 0.90
24. *Iodamoeba buetschlii* cysts: 0.90
25. *Paragonimus* spp. eggs: 0.90
26. *Schistosoma japonicum*/*mekongi* eggs: 0.85
27. *Schistosoma* *mansoni* eggs: 0.80
28. *Strongyloides* *stercoralis* larvae: 0.97
29. *Taenia* spp. eggs: 0.90
30. *Trichuris* *trichiura* eggs: 0.80

**4. Holdout metrics after threshold selection**:

Holdout precision-recall results from the Pramana scanner only, after applying thresholds. Correspond to a single point on the PR curve that was chosen

|  | Precision | Recall | Ground truths | True positives | False negatives | False positives |
| --- | --- | --- | --- | --- | --- | --- |
| *Ascaris lumbricoides*, fertile egg mamillated eggs | 0.955 | 0.875 | 24 | 21 | 3 | 1 |
| *Ascaris lumbricoides*, infertile egg mamillated eggs | 1 | 0.946 | 37 | 35 | 2 | 0 |
| *Balantioides coli* cyst | 0.96 | 1 | 24 | 24 | 0 | 1 |
| *Balantioides coli* trophozoites | 0.971 | 0.825 | 40 | 33 | 7 | 1 |
| *Blastocystis* spp. | 0.748 | 0.704 | 135 | 95 | 40 | 32 |
| *Paracapillaria philippinensis* egg | 1 | 0.971 | 35 | 34 | 1 | 0 |
| *Chilomastix mesnili* cyst | 0.792 | 0.864 | 44 | 38 | 6 | 10 |
| *Clonorchis*/*Opisthorchis* spp. eggs | 1 | 0.975 | 40 | 39 | 1 | 0 |
| *Cyclospora* spp. oocysts | 0.783 | 0.887 | 53 | 47 | 6 | 13 |
| *Cystoisospora belli* oocysts | 0.975 | 0.907 | 43 | 39 | 4 | 1 |
| Misc. small protozoans | 0.832 | 0.675 | 154 | 104 | 50 | 21 |
| *Endolimax nana* cysts | 0.82 | 0.683 | 60 | 41 | 19 | 9 |
| *Entamoeba* sp. (non-*hartmanni*) cysts | 0.875 | 0.843 | 83 | 70 | 13 | 10 |
| *Entamoeba* sp. (non-*hartmanni*) trophozoites | 0.828 | 0.828 | 58 | 48 | 10 | 10 |
| *Enterobius vermicularis* eggs | 1 | 0.947 | 19 | 18 | 1 | 0 |
| *Fasciola*/*Fasciolopsis* spp. Eggs | 1 | 1 | 25 | 25 | 0 | 0 |
| Fish tapeworm eggs | 1 | 1 | 23 | 23 | 0 | 0 |
| *Giardia duodenalis* cysts | 0.829 | 0.787 | 80 | 63 | 17 | 13 |
| *Giardia duodenalis* trophozoites | 0.816 | 0.674 | 46 | 31 | 15 | 7 |
| Hookworm/Trichostrongylus eggs | 0.976 | 0.952 | 42 | 40 | 2 | 1 |
| *Hymenolepis diminuta* eggs | 1 | 1 | 34 | 34 | 0 | 0 |
| *Rodentolepis nana* eggs | 1 | 0.919 | 37 | 34 | 3 | 0 |
| *Iodamoeba buetschlii* cysts | 0.804 | 0.932 | 44 | 41 | 3 | 10 |
| *Paragonimus* spp. eggs | 1 | 0.971 | 34 | 33 | 1 | 0 |
| *Schistosoma japonicum* eggs | 1 | 1 | 10 | 10 | 0 | 0 |
| *Schistosoma* *mansoni* eggs | 1 | 0.971 | 35 | 34 | 1 | 0 |
| *Strongyloides* *stercoralis* larvae | 1 | 0.976 | 42 | 41 | 1 | 0 |
| *Taenia* spp. eggs | 1 | 0.871 | 31 | 27 | 4 | 0 |
| *Trichuris* *trichiura* eggs | 1 | 1 | 28 | 28 | 0 | 0 |

**5 Work Flow Algorithm**

The following chart showed the workflow for accuracy and how discrepant scans were handled.


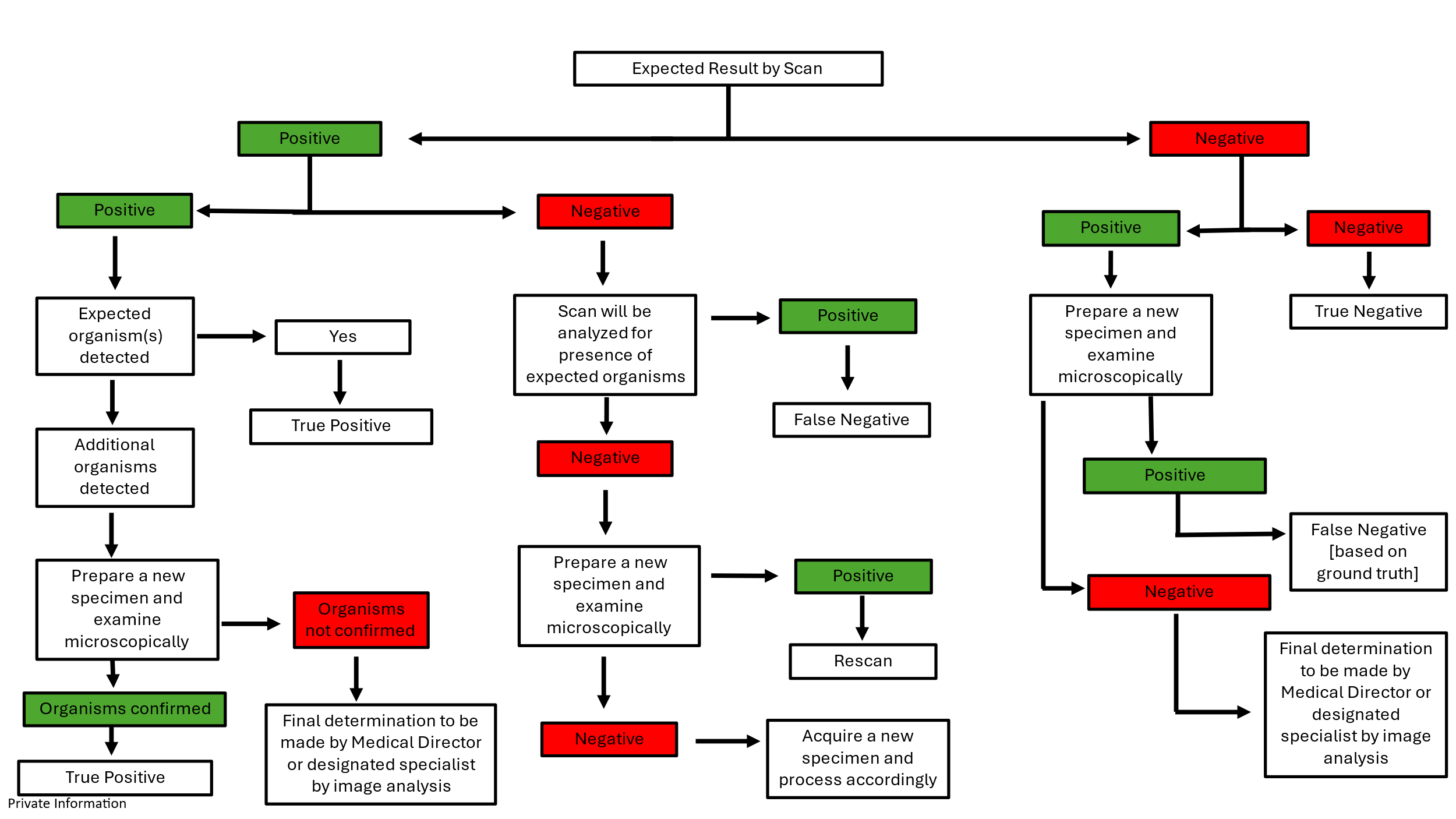


**6. Precision Data**

Within-run (same day) Reproducibility.

| Specimen | Scan 1 | Scan 2 | Scan 3 |
| --- | --- | --- | --- |
| TC-TAEN-6 | *Taenia* sp. (n=12) | *Taenia* sp. (n=3) | *Taenia* sp. (n=11) |
| ARUP-GD-2 | *Giardia duodenalis* (n=181) | *Giardia duodenalis* (n=61) | *Giardia duodenalis* (n=42) |
| ARUP-NEG-088 | Negative | Negative | Negative |

Between-run (multi-day) reproducibility

The three specimens (above) were prepared and scanned on three different days.

*Results*

All three scans detected the target organisms on three different days for the two positive specimens and the negative specimen was read as negative on all three days. The numbers for the positive species reflect both correct classification and class confusion. The variability in numbers is expected given the lack of homogeneity between wet mount preparations.

Between-run (multi-day) Reproducibility.

| Specimen | Day 1 | Day 2 | Day 3 |
| --- | --- | --- | --- |
| TC-TAEN-6 | *Taenia* sp. (n=12) | *Taenia* sp. (n=4) | *Taenia* sp. (n=10) |
| ARUP-GD-2 | *Giardia duodenalis* (n=181) | *Giardia duodenalis* (n=41) | *Giardia duodenalis* (n=72) |
| ARUP-NEG-088 | Negative | Negative | Negative |
